# Molecular Architecture of the ATP-Dependent Chromatin-Remodeling Complex SWR1

**DOI:** 10.1016/j.cell.2013.08.018

**Published:** 2013-09-12

**Authors:** Vu Q. Nguyen, Anand Ranjan, Florian Stengel, Debbie Wei, Ruedi Aebersold, Carl Wu, Andres E. Leschziner

**Affiliations:** 1 Department of Molecular and Cellular Biology, Harvard University, Cambridge, MA 02138, USA; 2 Laboratory of Biochemistry and Molecular Biology, Center for Cancer Research, National Cancer Institute, Bethesda, MD 20892, USA; 3 Department of Biology, Institute of Molecular Systems Biology, ETH Zurich, Zurich 8092, Switzerland; 4 Faculty of Science, University of Zurich, Zurich 8057, Switzerland; 5 HHMI Janelia Farm Research Campus, Ashburn, VA 20147, USA

## Abstract

The ATP-dependent chromatin-remodeling complex SWR1 exchanges a variant histone H2A.Z/H2B dimer for a canonical H2A/H2B dimer at nucleosomes flanking histone-depleted regions, such as promoters. This localization of H2A.Z is conserved throughout eukaryotes. SWR1 is a 1 megadalton complex containing 14 different polypeptides, including the AAA+ ATPases Rvb1 and Rvb2. Using electron microscopy, we obtained the three-dimensional structure of SWR1 and mapped its major functional components. Our data show that SWR1 contains a single heterohexameric Rvb1/Rvb2 ring that, together with the catalytic subunit Swr1, brackets two independently assembled multisubunit modules. We also show that SWR1 undergoes a large conformational change upon engaging a limited region of the nucleosome core particle. Our work suggests an important structural role for the Rvbs and a distinct substrate-handling mode by SWR1, thereby providing a structural framework for understanding the complex dimer-exchange reaction.

## Highlights

• SWR1 consists of four structurally discrete functional modules
• Rvb1 and Rvb2 assemble into a single, heterohexameric ring in SWR1
• SWR1 undergoes a large conformational change upon nucleosome binding
• SWR1 forms limited contact with the nucleosome core particle

## Graphical Abstract

**Figure:**
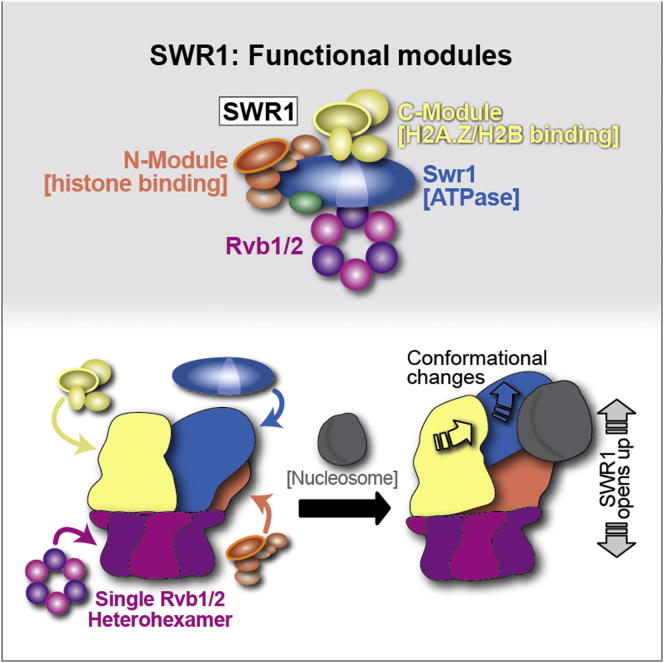

## Teaser

Structural analysis of the 14 subunit SWR1 chromatin remodeler identifies the orientation of its functional modules and reveals conformational changes induced by nucleosome binding.

## Introduction

Inside the nucleus, eukaryotic DNA condenses into chromatin by associating with evolutionarily conserved histone proteins H2A, H2B, H3, and H4. About 150 DNA base pairs wrap around a histone octamer, which comprises one (H3/H4)_2_ tetramer and two H2A/H2B dimers, to form the nucleosome (Luger et al., 1997). Essential nuclear activities are regulated by processes that target the nucleosome. These processes are best characterized at gene promoters, where the biophysical properties, position, and composition of nucleosomes are strictly regulated. This results in a stereotypical chromatin structure that includes a histone-depleted region flanked by labile yet well-positioned nucleosomes containing the evolutionarily conserved histone variant H2A.Z (Albert et al., 2007; Raisner et al., 2005; Tolstorukov et al., 2009). ATP-dependent chromatin remodeling complexes (remodelers) play a significant role in the regulation of promoter chromatin (Badis et al., 2008; Hartley and Madhani, 2009; Zhang et al., 2011).

Remodelers are conserved multisubunit complexes that can directly alter nucleosomal position and composition. All remodelers contain an ATPase domain—a member of the superfamily 2 (SF2) of translocases—within their core subunits (Clapier and Cairns, 2009). They also harbor domains located in *cis* to the ATPase that can regulate its ATPase activity (Clapier and Cairns, 2012; Hota and Bartholomew, 2011) and bind accessory subunits (Szerlong et al., 2008) and/or histone modifications (Clapier and Cairns, 2009). These auxiliary domains are the basis for the common classification of remodelers into four subfamilies: SWI/SNF, ISWI, CHD, and INO80 (Clapier and Cairns, 2009). Many remodelers collaborate at gene promoters to regulate transcriptional competency. Complexes of the SWI/SNF and ISWI subfamilies establish a nucleosome-depleted region around the promoter, thus exposing it to the transcriptional machinery (Clapier and Cairns, 2009). SWR1, a member of the INO80 subfamily, is targeted to this region to deposit H2A.Z at flanking nucleosomes (Hartley and Madhani, 2009; Kobor et al., 2004; Krogan et al., 2003; Venters and Pugh, 2009). H2A.Z has been shown to affect the stability of its host nucleosome (Park et al., 2004; Suto et al., 2000), higher-order chromatin folding (Fan et al., 2002, 2004), and recruitment of transcriptional factors (Draker et al., 2012).

**Figure 1:**
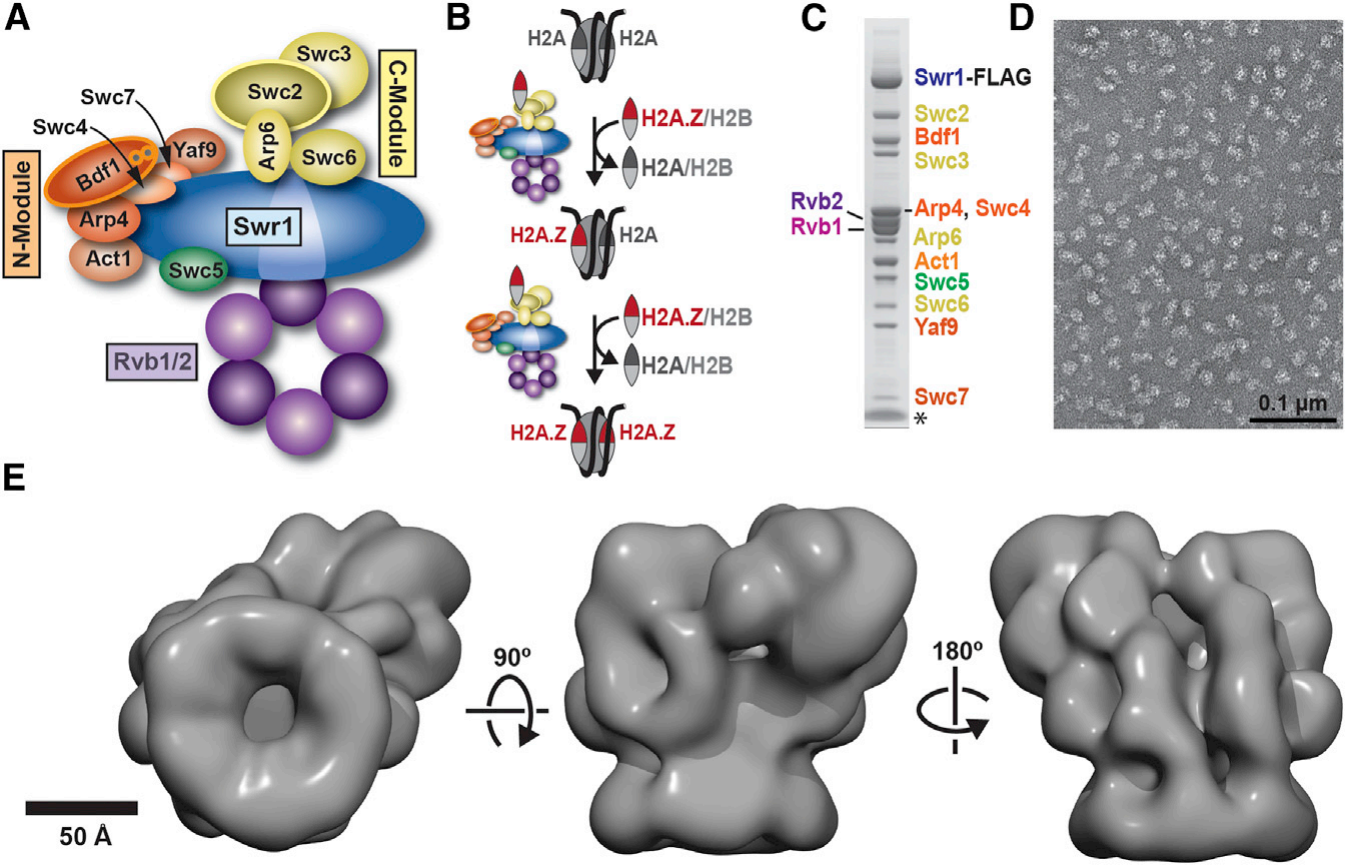
3D Reconstruction of the SWR1 Complex (A) Schematic representation of the SWR1 complex. The arrangement of its 14 subunits is based on previous studies. (B) Schematic representation of the histone dimer exchange catalyzed by SWR1. (C) SDS-PAGE of SWR1 affinity purified from *S. cerevisiae*. The 14 subunits, including FLAG-tagged Swr1, are indicated and color coded as in (A). The band at the bottom of the gel (^∗^) is the 3× FLAG peptide used to elute the complex from the affinity resin. (D) Electron micrograph of cryo-NS SWR1 after further stabilization and purification through a GraFix gradient (see text). (E) Cryo-NS structure of SWR1 at 28 Å resolution. A hexameric feature can be seen in the leftmost view of the complex. See also Figures S1 and S2, Table S1, and Movie S1.

Most remodelers can reposition the nucleosome by “sliding” the histone octamer along the DNA. Although this activity depends on ATP-dependent DNA translocation by the core ATPase (Saha et al., 2002, 2005; Zofall et al., 2006), remodelers function as multisubunit complexes (Clapier and Cairns, 2009). This highlights the importance of understanding how functional components assemble together into a remodeling machine and how this machine engages the nucleosome substrate. Structural approaches aimed at answering these questions are limited in number and resolution due to the complex compositions (2–15 subunits) and relatively large sizes (200–1,400 kDa) of remodelers (Leschziner, 2011). Nevertheless, they have provided some significant mechanistic insights. For example, a three-dimensional electron microscopy (3D EM) structure of the RSC complex (Chaban et al., 2008) showed it enveloping the nucleosome within a central cavity. The structure of nucleosome-bound ACF revealed that two remodelers bind to one nucleosome (Racki et al., 2009), which may underlie its ability to “measure” linker DNA and generate arrays of evenly spaced nucleosomes. In contrast to these “sliders,” SWR1 has evolved a mechanism for dimer exchange (Luk et al., 2010; Mizuguchi et al., 2004), which involves ejecting a resident H2A/H2B dimer from the substrate nucleosome and inserting a H2A.Z/H2B dimer in its place (Figure 1B).

SWR1 functions as an ∼1 MDa complex containing 14 different polypeptides. Detailed dissection of its composition revealed three multisubunit modules that assemble on the core, catalytic subunit Swr1 (Figure 1A) (Wu et al., 2005, 2009). The N-terminal half of the Swr1 polypeptide contains the helicase-SANT-associated (HSA) domain, which interacts with nuclear actin-related proteins (Arps) (Szerlong et al., 2008). The Bdf1-Arp4-Act1-Swc4-Yaf9-Swc7 module—referred to as the N-Module here—is recruited to this region (Wu et al., 2005). Arp4 has been shown to interact directly with canonical nucleosomes and histones (Galarneau et al., 2000), Swc4 contains a SANT domain implicated in binding unmodified histone tails (Boyer et al., 2004), and Bdf1 contains tandem bromodomains with affinity for acetylated histone tails (Jacobson et al., 2000; Pamblanco et al., 2001). Therefore, the N-Module is likely involved in the targeting and binding of SWR1 to hyperacetylated nucleosomes. The core ATPase domain resides in the C-terminal half of Swr1 and harbors within it a long insert domain that is characteristic of the INO80, or “split-ATPase,” subfamily. This insert facilitates association of the two remaining modules, Swc3-Swc2-Arp6-Swc6 (termed here the C-Module) and the putative hexameric helicases Rvb1 and Rvb2 (Wu et al., 2005), the latter module being another distinguishing characteristic of the INO80 subfamily. The C-Module binds the H2A.Z/H2B dimer, which is to be incorporated into the nucleosome, via the Swc2 subunit (Wu et al., 2005). Finally, Rvb1 and Rvb2 are AAA+ ATPases that can form homohexameric (Matias et al., 2006), heterohexameric (Gribun et al., 2008), or dodecameric ring structures (Cheung et al., 2010a, 2010b; Puri et al., 2007; Torreira et al., 2008) in isolation. Their oligomeric state in large complexes is not known structurally, although it has been suggested that they exist as a dodecamer, or double rings, in the SWR1-related INO80 complex (Kapoor et al., 2013; Shen et al., 2000).

To address how functional modules of a dimer exchanger assemble as a complex, we have undertaken a multipronged approach to characterize the molecular architecture of SWR1 from *Saccharomyces cerevisiae*. Using EM, we have determined the 3D structure of SWR1 and mapped the locations of all functional modules. Our results show that the substrate-handling N- and C-Modules are arranged side-by-side and bracketed by the Swr1 ATPase and a single hexameric Rvb1/Rvb2 ring. Furthermore, neighboring relationships within SWR1, determined by chemical crosslinking and mass spectrometric (CX-MS) analysis (Leitner et al., 2010), show that its components are highly interconnected. Finally, our reconstruction of a SWR1-nucleosome cocomplex reveals a large conformational change in the enzyme, which only forms limited contacts with the nucleosome substrate. Our data provide a structural framework for understanding SWR1’s unique dimer-exchange activity.

## Results

### 3D Reconstruction of SWR1

**Figure S1:**
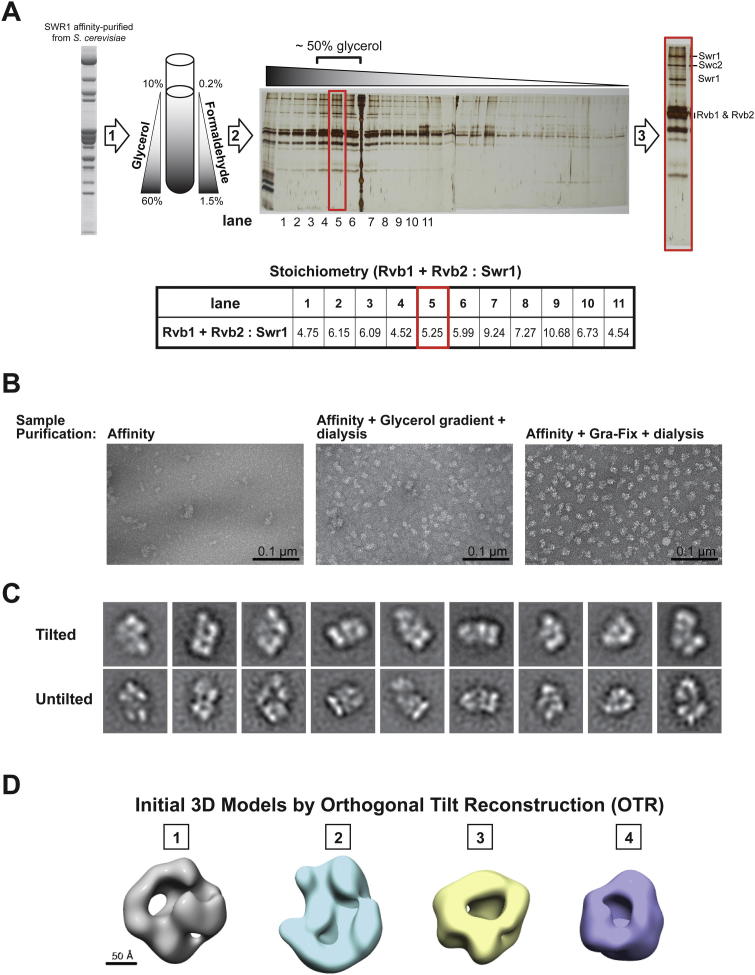
Purification of SWR1 by GraFix and Initial Model Generation Using Orthogonal Tilt Reconstruction, Related to Figures 1 and 2 (A) Native SWR1 was affinity-purified from *S. cerevisiae* as described (Luk et al., 2010) (1) The affinity-purified sample was run through a GraFix gradient (see main text for details) (Kastner et al., 2008). (2) Upon fractionation of the gradient, small aliquots from all the fractions were treated to reverse the formaldehyde crosslinks and then analyzed by SDS-PAGE. (3) After the main SWR1 peak was identified, the peak fractions were further inspected in the electron microscope using negative stain. The SDS-PAGE lane to the right represents the fraction used to prepare grids for data collection and initial model generation; crosslinks are only reversed for analytical purposes and not in the imaged samples. The relative amounts of the Swr1 subunit and the Rvb’s (Rvb1 + Rvb2) were measured for the different fractions in the main SWR1 peak and are indicated in the table below the gel. (B) Electron micrographs showing the appearance of the negative stained SWR1 sample after different purification strategies: affinity purification (left); affinity purification → glycerol gradient → dialysis (center); and affinity purification → GraFix → dialysis (right). The dialysis reduces the high glycerol concentration in the sample, which would otherwise affect the staining. (C) 2D class averages obtained from data collected at (±) 45° tilt exhibit similarity to those obtained from untilted data (Chandramouli et al., 2011). (D) Initial 3D models obtained using Orthogonal Tilt Reconstruction (Leschziner, 2010).

We used 3D EM to determine the structure of SWR1 obtained from *S. cerevisiae*. Although the affinity-purified sample appeared biochemically pure, with all 14 SWR1 components and no visible contaminants (Figure 1C), it was structurally heterogeneous when observed under the electron microscope (Figure S1B available online). To overcome this, we adapted the GraFix (*Gra*dient with *Fix*ation) technique, which uses a combined glycerol and crosslinker gradient (Figure S1A) to both stabilize and purify macromolecular assemblies (Kastner et al., 2008; Stark, 2010). We chose formaldehyde as the crosslinker because reversibility of the crosslinks would allow us to verify where in the gradient all SWR1 components were present (Figure S1A). We then imaged individual fractions to identify the most homogeneous sample for data collection (Figure 1D).

**Figure S2:**
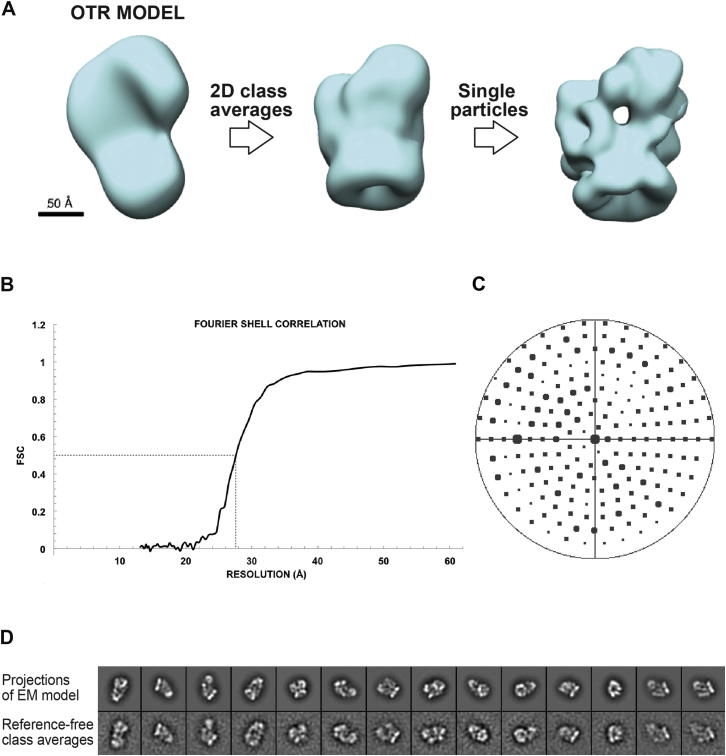
Refinement of OTR Initial Model Using Cryo-Negative Stain Data, Related to Figures 1 and 2 (A) The OTR initial model (left) was first refined by projection matching against reference-free class averages generated from untilted, cryo-negative stain (cryo-NS) images of SWR1. The resulting structure (middle) was further refined by projection matching against individual cryo-NS images of SWR1. The final refined structure, filtered to 28 Å, is shown to the right. (B) Fourier Shell Correlation calculated for the final refined structure. The frequencies are shown as resolution, its inverse, in this graph. (C) Angular distribution of the images used for refinement. The position of each circle in the plot represents the set of Euler angles corresponding to one reference image using during projection matching. The radius of the circle is proportional to the number of particles assigned to that particular reference. (D) Comparison between reprojections of the SWR1 3D map and experimental reference-free class averages.

We obtained initial, low-resolution models of SWR1 using the Orthogonal Tilt Reconstruction (OTR) approach (Leschziner, 2010) and negatively stained samples (Figures S1C and S1D). To refine the models, we used cryo-negative stain (cryo-NS) data, which benefit from the high contrast provided by the heavy-atom stain and the structural integrity of frozen-hydrated samples (De Carlo and Stark, 2010). We performed projection-matching refinement, first against reference-free class averages and then against single particles (Figure S2A). The resulting 3D structure (Figure 1E; Movie S1) has an estimated resolution of 28 Å (0.5 Fourier Shell Correlation [FSC]) (Figure S2B). Two-dimensional (2D) projections calculated from this structure show a good match to reference-free class averages obtained from the cryo-NS data (Figure S2D).

### The SWR1 Complex Contains a Single Heterohexameric Rvb1/Rvb2 Ring

**Figure 2:**
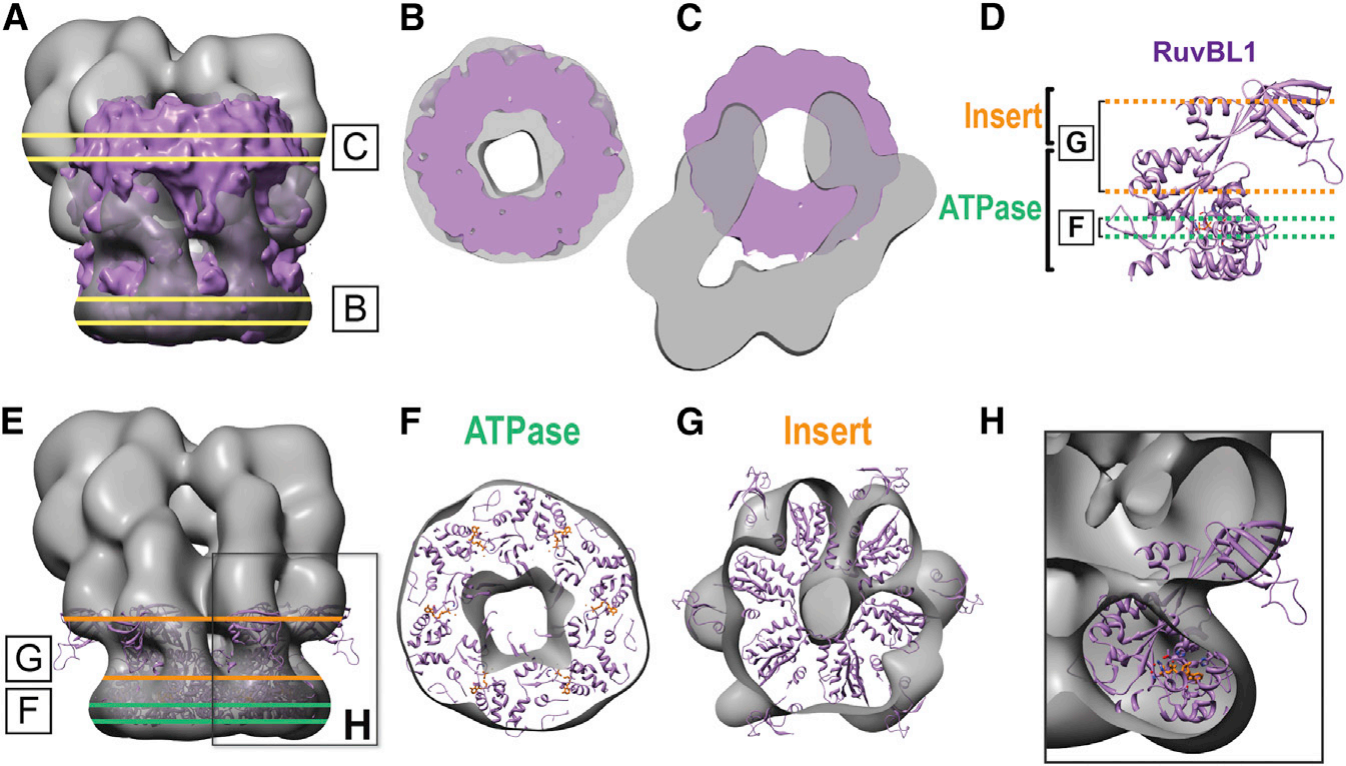
Rvb1 and Rvb2 Assemble as a Single Heterohexameric Ring in the SWR1 Complex (A) The 13 Å cryo-EM structure of an Rvb1/Rvb2 dodecamer from yeast (EMD-2865) (Torreira et al., 2008) was superimposed on the cryo-NS map of the SWR1 complex (this work). The two slices shown in (B) and (C) are indicated. (B and C) Views perpendicular to slices B (B) and C (C) show the contours of the Rvb1/Rvb2 (purple) and SWR1 (gray) maps. (D) The ATPase and insert domains are labeled on the crystal structure of the human ortholog of Rvb1 (RuvBL1) (Protein Data Bank ID code [PDB] 2C9O) (Matias et al., 2006). The portions of the structure visible in (F) and (G) are indicated. (E) The crystal structure of the RuvBL1 hexamer (PDB 2C9O) was docked into the hexameric density at the bottom of the SWR1 cryo-NS map. The two slices shown in (F) and (G) and the region analyzed in (H) are indicated. (F) View perpendicular to slice F, corresponding to the ATPase domains. (G) View perpendicular to slice G, corresponding to the inserts and a portion of the ATPase immediately adjacent to them. (H) The front half of the EM density was removed to show a single RuvBL1 monomer docked into the SWR1 EM density map. The orientation of the RuvBL1 structure is identical to that shown in (D). See also Figure S3.

**Figure 3:**
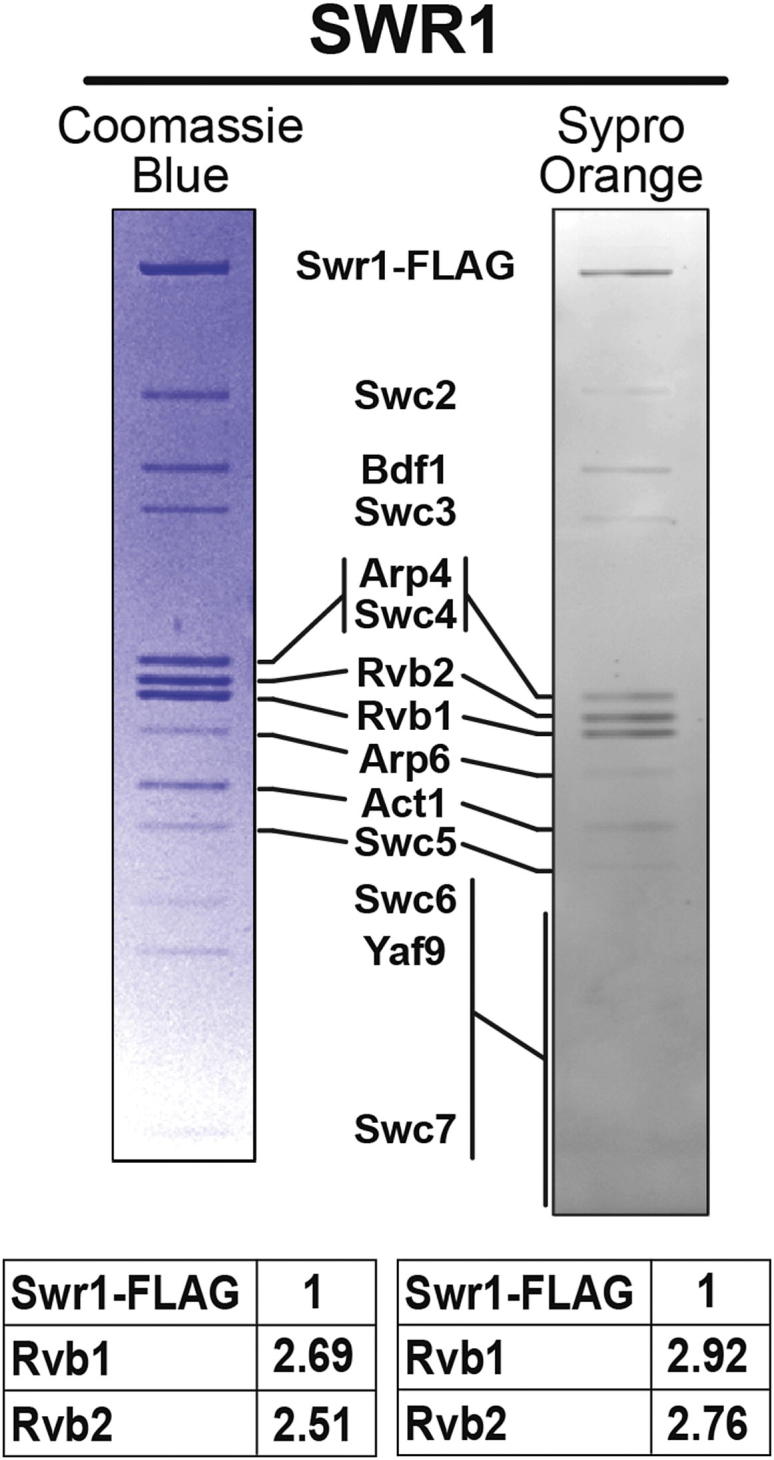
SWR1 Contains Three Copies Each of Rvb1 and Rvb2 SWR1 was affinity purified and resolved on 4%–12% Bis-Tris gels. Gels were stained either with Coomassie blue (left) or SYPRO Orange (right) and digitized. The intensities of the Rvb1 and Rvb2 bands, relative to Swr1-FLAG, are shown in the tables below the gels. See also Figure S4.

**Figure S3:**
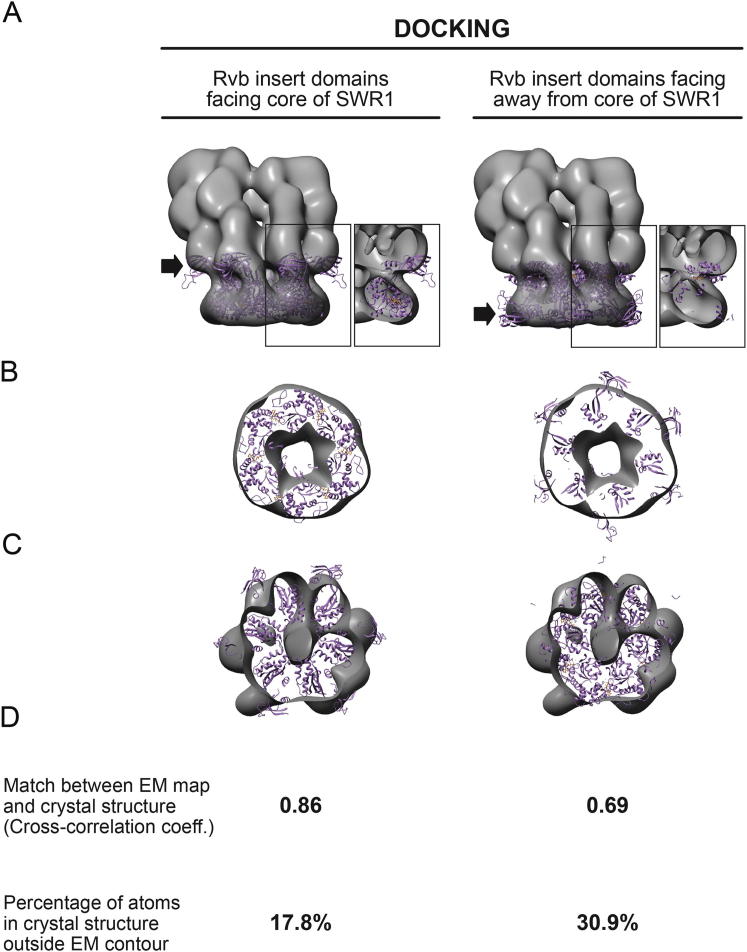
Docking of the Crystal Structure of the RuvBL1 Hexamer into the SWR1 EM Map, Related to Figure 2 The RuvBL1 structure (PDB: 2C9O) (Matias et al., 2006) was docked into the SWR1 cryo-NS map in two possible orientations: with the RuvBL1 insert domains pointing up, toward the core of SWR1 (left) or down, away from the core of SWR1 (right). The docking shown on the left is the one used throughout the main figures of the paper. (A) View of the full SWR1 map as a semi-transparent surface with the hexamer docked in. The insets show the region highlighted by the rectangle with the front half of the SWR1 contour removed and a single RuvBL1 monomer displayed. These insets are equivalent to (H) in Figure 2. The black arrow points to the position of the insert domains, which are known to be responsible for mediating ring-ring interactions in all available structures of stacked-ring arrangements of Rvb1/2 or their orthologs (Cheung et al., 2010a; Gorynia et al., 2011; López-Perrote et al., 2012; Torreira et al., 2008). (B) Slice through the volumes in A, equivalent to (F) in Figure 2. (C) Slice through the volumes in A, equivalent to (G) in Figure 2. (D) Quantitation of the fits shown in A. Top: the cross-correlation coefficient is calculated between the experimental SWR1 density and a density calculated at the same resolution (28Å) from the crystal structure (as implemented in UCSF Chimera). Bottom: the percentage of atoms in the RuvBL1 structure found outside of the SWR1 contour.

**Figure S4:**
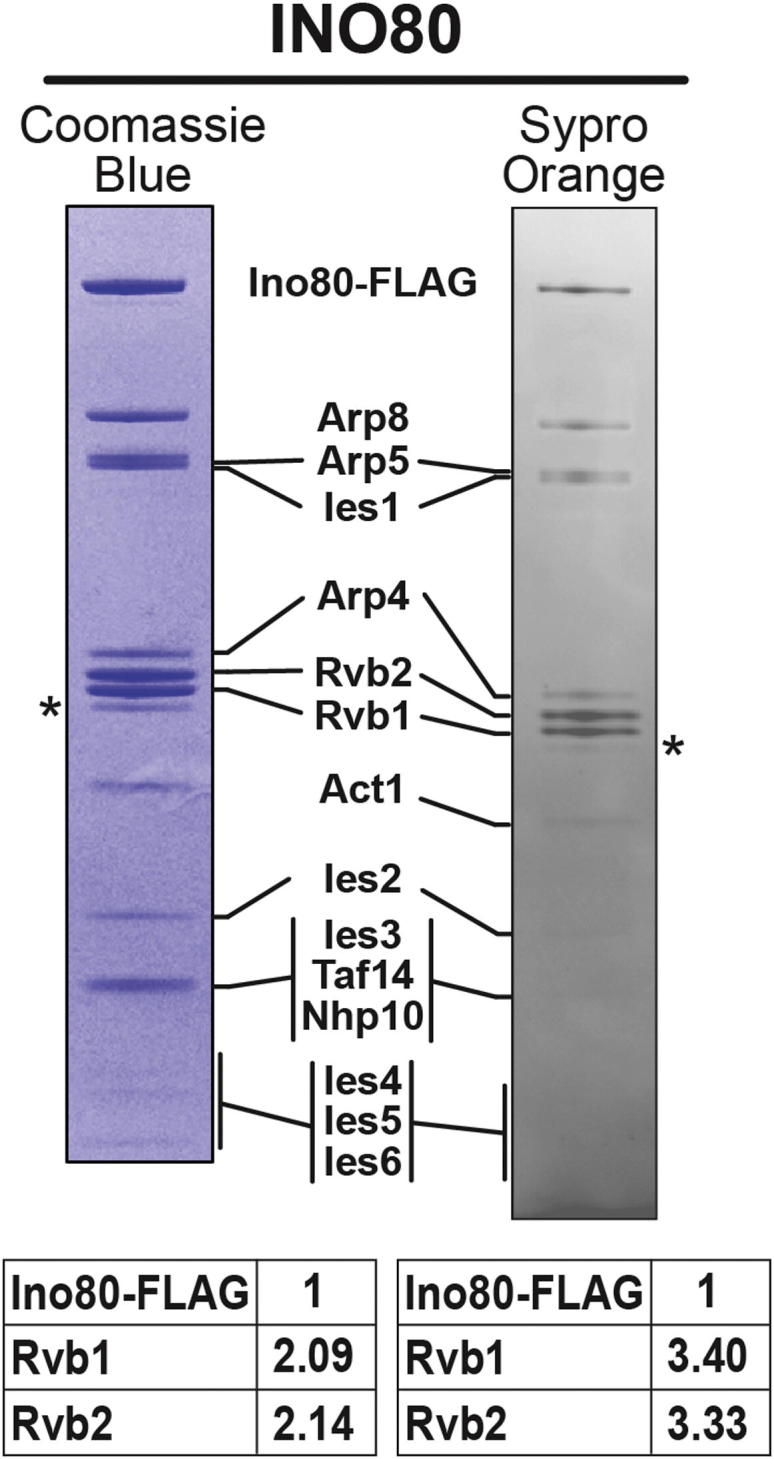
Stoichiometry Measurements of Rvb1, Rvb2, and Ino80, Related to Figure 3 Affinity and glycerol gradient purified INO80 complex was resolved on 4%–12% Bis-Tris gels (Invitrogen) and run with 1X MOPS buffer. Under these conditions the two Rvb proteins are well separated and an un-identified protein is observed right below Rvb1 (^∗^). This extra band, when not resolved from Rvb1 and Rvb2, might have contributed to a different Ino80:Rvb1/2 ratio reported in previous studies (Kapoor et al., 2013; Shen et al., 2000).

Our 3D map of SWR1 shows a clear ring-shaped density with hexameric features (Figure 1E, left, and Figure 2), which fits well the crystal structure of a hexamer of RuvBL1 (Matias et al., 2006), the human ortholog of Rvb1 (Figures 2D–2H). The orientation in our docking indicates that the Rvb insert domains (Figure 2D) mediate the ring’s interaction with the core of the SWR1 complex. Docking the RuvBL1 in the opposite orientation, with the insert domains facing away from the core of SWR1, results in a much poorer fit (Figure S3). The flexible Rvb insert domains (López-Perrote et al., 2012) protrude from the EM map (Figures 2G and 2H), suggesting that they undergo a rearrangement in SWR1. We also attempted to dock a 13 Å cryo-EM structure of a Rvb1/Rvb2 dodecamer from *S*. *cerevisiae* (Torreira et al., 2008) into our structure, but only one of its two rings fits the SWR1 density well (Figures 2A–2C). We further validated our structural data by performing a quantitative analysis of the Swr1:Rvb1:Rvb2 stoichiometry, which we determined to be ∼1:3:3 in the affinity-purified sample (Figure 3) and GraFix-treated fractions (Figure S1A), consistent with the presence of a single hexameric ring in SWR1. This stoichiometry does not appear to be a feature unique to SWR1 because we obtained similar results for the related INO80 complex (Figure S4).

Although both Rvb1 and Rvb2 copurify with Swr1 (Mizuguchi et al., 2004), it was formally possible that two populations of SWR1 coexist, each containing a homohexameric ring of either Rvb1 or Rvb2. Using CX-MS (Leitner et al., 2010), we identified a crosslink between Rvb1 and Rvb2 (Figure 5; Table S2). All homotypic (Rvb1-Rvb1 or Rvb2-Rvb2) crosslinks we identified corresponded to distances most compatible with intramolecular crosslinks (Table S2), whereas the Rvb1-Rvb2 crosslink agrees with the expected intersubunit interface based on a homology model of the *S*. *cerevisiae* Rvb1 and Rvb2 heterohexamer (Figures 5B and 5C). Thus, we conclude that SWR1 contains a single Rvb1/Rvb2 heterohexamer.

### The N- and C-Modules Form Discrete Structural Entities Bracketed by Rvb1/Rvb2 and the Swr1 ATPase

**Figure 4:**
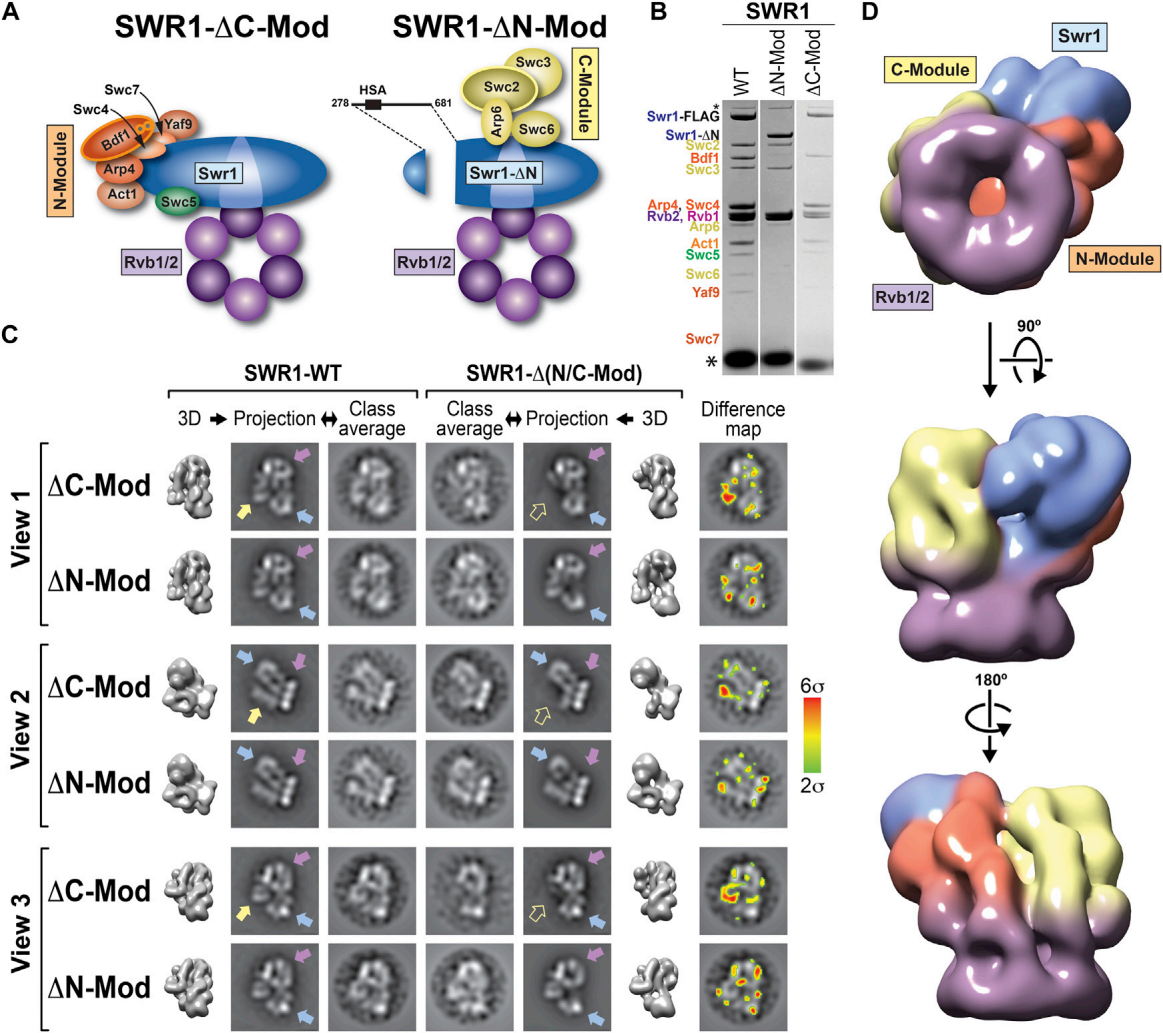
Two Functional Modules of SWR1 Assemble as Discrete Structural Entities Sandwiched between Rvb1/Rvb2 and the Swr1 ATPase (A) Schematic representations of the two subcomplexes used for our analysis, SWR1-ΔN-Mod and SWR1-ΔC-Mod. Both subcomplexes contain the Swr1 ATPase and Rvb1/Rvb2 but differ in the presence or absence of subsets of subunits, termed the N- and C-Modules, based on the portion of Swr1 with which they are known to interact (Wu et al., 2009). (B) SDS-PAGE analysis of SWR1-ΔN-Mod and SWR1-ΔC-Mod shows their compositions. The band at the bottom of the gels (^∗^) is the 3× FLAG peptide used to elute the complex from the affinity resin. Both samples were further purified and stabilized through a GraFix gradient before imaging (see text). (C) 2D image analysis of subcomplexes and mapping of N- and C-Modules are shown for three characteristic views of SWR1 (Views 1, 2, and 3). For each view, both SWR1-ΔC-Mod (top) and SWR1-ΔN-Mod (bottom) were analyzed. Each row shows, from left to right, (i) the 3D SWR1 structure in the orientation corresponding to that particular view; (ii) a reprojection from the SWR1 structure; (iii) a reference-free class average matching the reprojection; (iv) the corresponding reference-free class average for the subcomplex; (v) a reprojection from a 3D model of SWR1 where the N- or C-Module was digitally removed; (vi) the 3D model of SWR1 used to generate the reprojection in (v); and (vii) a difference map calculated by subtracting the class average of the subcomplex (iv) from that of full SWR1 (iii). The difference map is colored according to the scale shown to the right and is overlaid on top of the class average of full SWR1. The purple and blue arrows point to those structures that are present in both SWR1 and the subcomplexes and are color coded according to the final assignment of molecular identities shown in (D). The yellow arrows point to large features that are present in SWR1 (solid arrow) but absent in SWR1-ΔC-Mod (hollow arrow). (D) The same three views of SWR1 shown in Figure 1E are now color coded according to the identities of the four functional modules: Swr1, Rvb1/Rvb2, N-Module, and C-Module. See also Figure S5 and Table S1.

**Figure S5:**
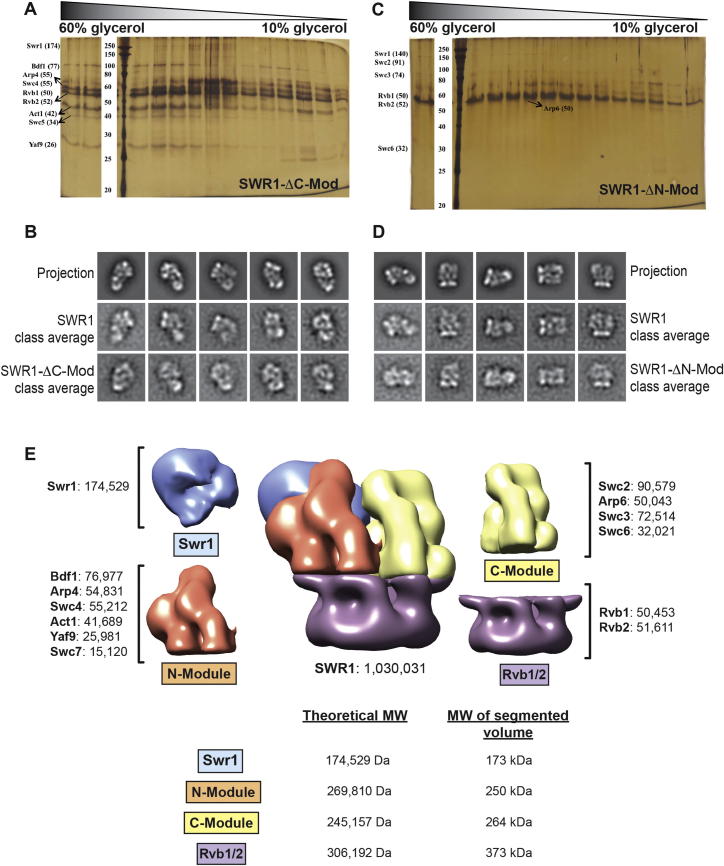
Mapping Functional Models by Structural Analysis of Stable SWR1 Subcomplexes, Related to Figure 4 (A) Stabilization and purification of a SWR1 sub-complex missing the C-Module (SWR1-ΔC-Mod) by GraFix. (B) Comparison between experimental reference-free class averages generated from cryo-NS images of SWR1-ΔC-Mod and the corresponding reprojections and class averages of full SWR1. (C) Stabilization and purification of a SWR1 sub-complex missing the N-Module (SWR1-ΔN-Mod) by GraFix. (D) Comparison between experimental reference-free class averages generated from cryo-NS images of SWR1-ΔN-Mod and the corresponding reprojections and class averages of full SWR1. (E) The 3D EM map of SWR1 was segmented according to the module boundaries approximated in Figure 4. The subunits present in each of the four functional modules are indicated along with their molecular weights. The table at the bottom shows the theoretical MW obtained from adding the subunits in each module and the calculated MW obtained from the number of voxels enclosed by each segmented density.

To determine the locations of the N- and C-Modules in the SWR1 structure, we characterized stable subcomplexes containing Swr1 and Rvb1/Rvb2, and either the N- or C-Module (Wu et al., 2009) (Figures S5A and S5C; Table S1). We named these ∼700 kDa subcomplexes SWR1-ΔC-Mod and SWR1-ΔN-Mod to indicate the missing module (Figures 4A and 4B).

We aligned and classified cryo-NS images to obtain 2D class averages, or views, of the subcomplexes. A comparison of these views with those from the full complex and 2D reprojections of the SWR1 structure led to two major observations. First, in the absence of either ∼300 kDa module, the remaining SWR1 components assembled into a structure very similar to that of the corresponding portion of the full complex (Figures 4C, S4B, and S4D). Notably, an ∼400 amino acid truncation in Swr1 did not significantly impact the assembly of the remaining subunits in SWR1-ΔN-Mod, suggesting that the N- and C-terminal halves of Swr1 fold independently of each other. Second, two major features were retained in both subcomplexes: the Rvb1/Rvb2 ring, and a prominent density distal to it (Figures 4C, S4B, and S4D). Because the subcomplexes only share Rvb1, Rvb2, and the catalytic bulk of Swr1, we conclude that the latter occupies the large density distal to the Rvb1/Rvb2 ring (Figure 4D).

Next, we performed difference mapping between class averages of each subcomplex and those of full SWR1 for three different views of (Figure 4C). This analysis identified the locations of the C- and N-Modules. They form structurally discrete entities arranged side-by-side and bracketed by the Rvb1/Rvb2 ring and Swr1 (Figures 4C and 4D). These results allowed us to generate a low-resolution annotation of the 3D map (Figure 4D). Further support for this annotated map was provided by the general agreement of the theoretical molecular weights of the modules with those calculated from the corresponding densities in the EM map (Figure S5E).

### Isotopic Crosslinking-Mass Spectrometry Maps Subunit Arrangement within SWR1

**Figure 5:**
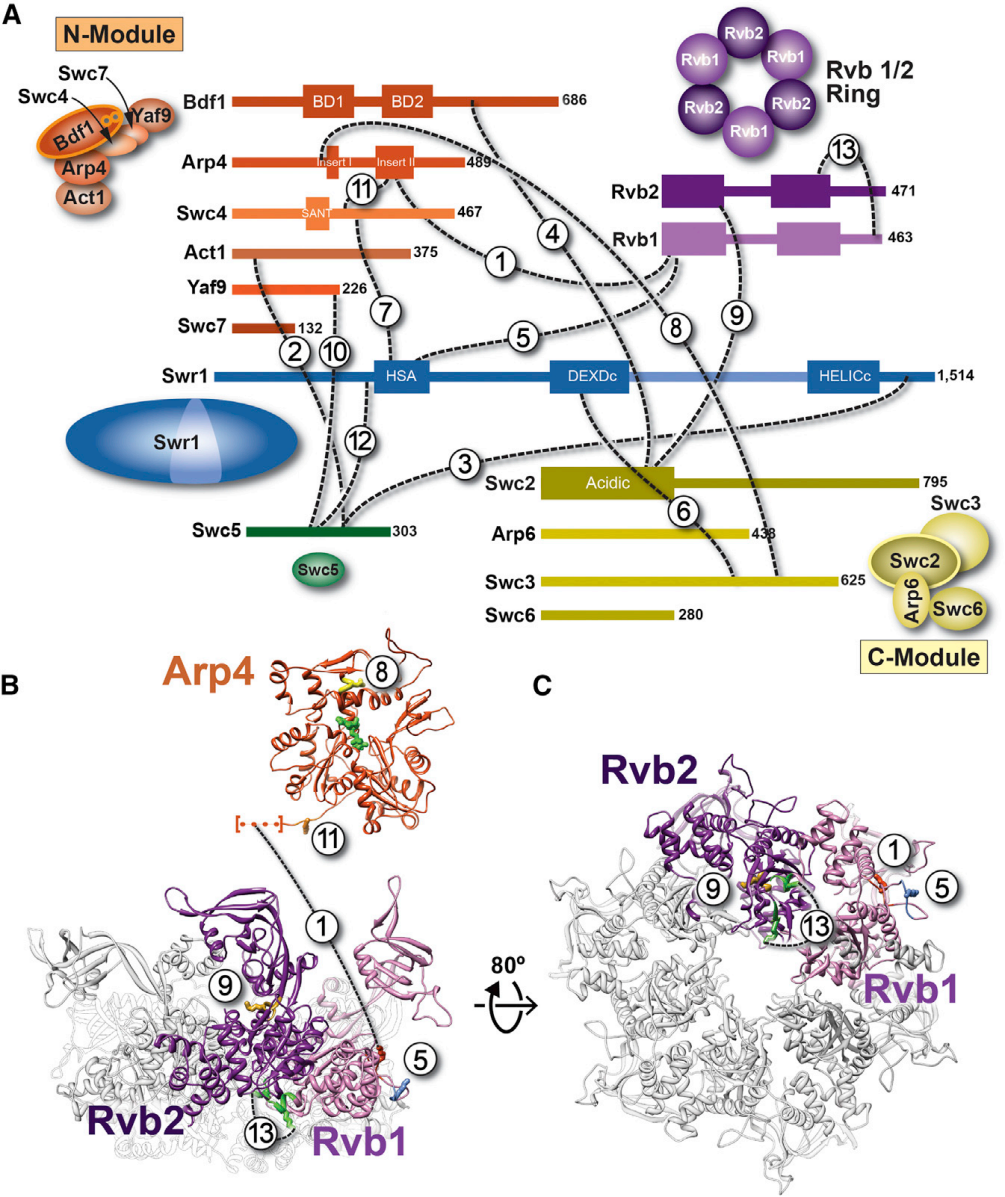
Isotopic CX-MS Analysis of the SWR1 Complex (A) Schematic representation of interprotein crosslinks detected by CX-MS analysis of SWR1. The 14 polypeptides in SWR1 are color coded according to the diagram shown in Figure 1A and are drawn proportionally to their mass. The number of amino acids in each polypeptide is indicated to its right. The SWR1 subunits are grouped into the N-Module, C-Module, Rvb1/Rvb2 ring, catalytic subunit Swr1, and Swc5 subunit. Select crosslinks identified in this study are shown with dashed black lines and are numbered (1–13). (B) Mapping of the crosslinks involving Rvb1, Rvb2, and Arp4 to their crystal structures. The Arp4 structure is that of the *S*. *cerevisiae* protein (3QB0) (Fenn et al., 2011), whereas the Rvb1/Rvb2 heterohexamer is a homology model generated for this study using the crystal structure of the human ortholog RuvBL1 (2C9O) (Matias et al., 2006) (see Extended Experimental Procedures). The crosslinked lysines are shown as sticks and color coded according to the crosslink partner shown in (A) (except for the residues involved in the Rvb1-Rvb2 crosslink, which are colored green). The numbers placed next to the crosslinked lysines refer to the crosslink numbers shown in (A). The dotted line within square brackets in Arp4 indicates the location of an observed crosslink that maps to a portion of the sequence not resolved in the crystal structure. (C) A view of the Rvb1/Rvb2 ring perpendicular to its plane shows the locations of the identified crosslinks. See also Table S2.

We used isotopic CX-MS (Leitner et al., 2010) to determine the spatial organization of SWR1 subunits in more detail. Our results confirmed a number of the previously determined interactions (Figure 5; Table S2). Specifically, we observed crosslinks connecting the N-Module with the N-terminal half of Swr1 (Swc4-Swr1), the C-Module with the C-terminal half of Swr1 (Swc3-Swr1), and Arp4 with Swc4 within the N-Module (Figure 5A; Table S2). Additionally, we obtained crosslinks connecting the small subunit Swc5 with both the N- and C-terminal halves of Swr1, in agreement with data showing that Swc5 requires the full SWR1 polypeptide to be present in the complex (Wu et al., 2009).

We also observed additional crosslinks that suggest a high degree of interconnectedness among SWR1’s functional modules. The Rvb1/Rvb2 ring, previously shown to require the long insert in the ATPase domain of Swr1 to assemble into the full complex (Wu et al., 2005), crosslinked to the N-terminal half of Swr1 (its HSA domain) via Rvb1, the N-Module (Rvb1-Arp4), and the C-Module (Rvb2-Swc2) (Figure 5; Table S2). The N- and C-Modules also crosslinked to each other, through Bdf1-Swc2 and Arp4-Swc3 (Figure 5A; Table S2). Finally, Swc5 crosslinked to the N-Module via two interactions with Act1 and Yaf9 (Figure 5A; Table S2). Understanding the functional significance of these novel spatial relationships will require future work combining biochemistry and finer subunit mapping in SWR1.

### SWR1 Adopts an Extended Conformation in the Presence of a Nucleosome

**Figure 6:**
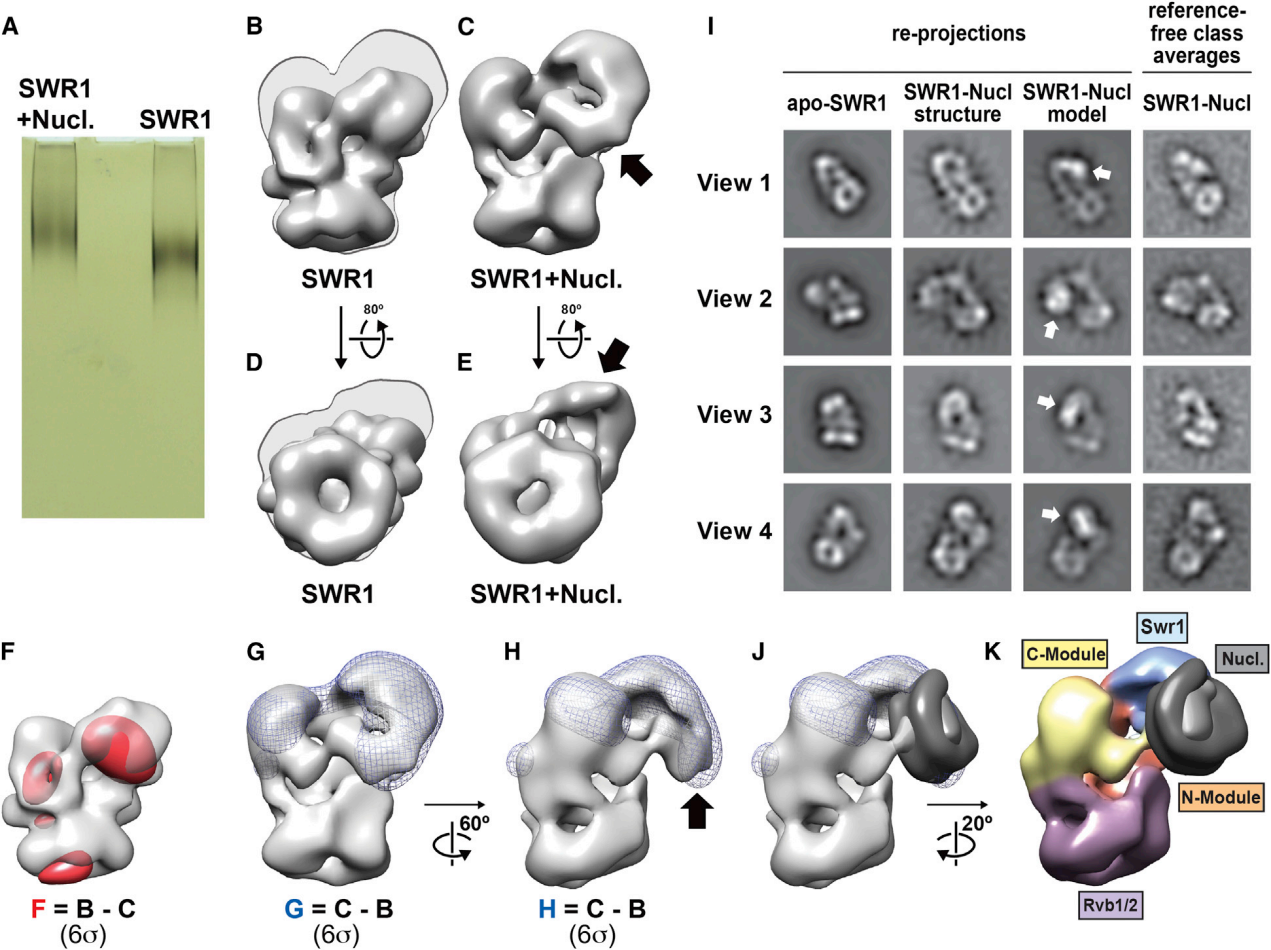
SWR1 Undergoes a Conformational Change in the Presence of a Nucleosome (A) Native PAGE of GraFix-stabilized SWR1 + nucleosome (SWR1+Nucl.; left) and SWR1 alone (SWR1; right). (B) The structure of SWR1 (apo-SWR1) filtered to 34 Å resolution. (C) 3D reconstruction of a SWR1-nucleosome complex at a resolution of 34 Å. The shadow shown in (B) is the silhouette of the structure in (C) to highlight the overall elongation of the structure. (D and E) The structures in (B) and (C) are seen from the Rvb1/Rvb2 ring. The silhouette of the structure in (E) is shown behind the structure in (D). The arrows in (C) and (E) point to new densities visible in the SWR1-nucleosome reconstruction. (F and G) Difference maps were obtained by subtracting (F) SWR1-nucleosome from apo-SWR1 (red densities, superimposed on apo-SWR1) or (G) apo-SWR1 from SWR1-nucleosome (blue mesh, superimposed on SWR1-nucleosome). The structures were filtered to 60 Å before calculating the difference maps. The difference maps were contoured to 6σ and represent either those parts of the structure present in apo-SWR1 but absent in SWR1-nucleosome (F) or present in SWR1-nucleosome but absent in apo-SWR1 (G). (H) A side view of the structure in (G) shows the superposition between the new density and a peak in the difference map (black arrow). (I) 2D image analysis of SWR1-nucleosome data for four different views of the complex. The panel shows, from left to right (i) reprojections of apo-SWR1 that best match the view of SWR1-nucleosome analyzed; (ii) the corresponding reprojections of the SWR1-nucleosome reconstruction; (iii) the corresponding reprojections of the SWR1-nucleosome 3D model shown in (J) (the white arrows point to the nucleosome); and (iv) the corresponding reference-free class averages from the SWR1-nucleosome data. (J) 3D model for the SWR1-nucleosome complex. A nucleosome, filtered to 34 Å, was placed in the peak in the difference map based on the 2D image analysis shown in (I). The nucleosome is shown in gray with the H2A/H2B dimer in a lighter shade. (K) The SWR1-nucleosome model shown in (J), is color coded according to the identities of the four functional modules. The nucleosome is colored as in (J). See also Figures S6 and S7.

**Figure S6:**
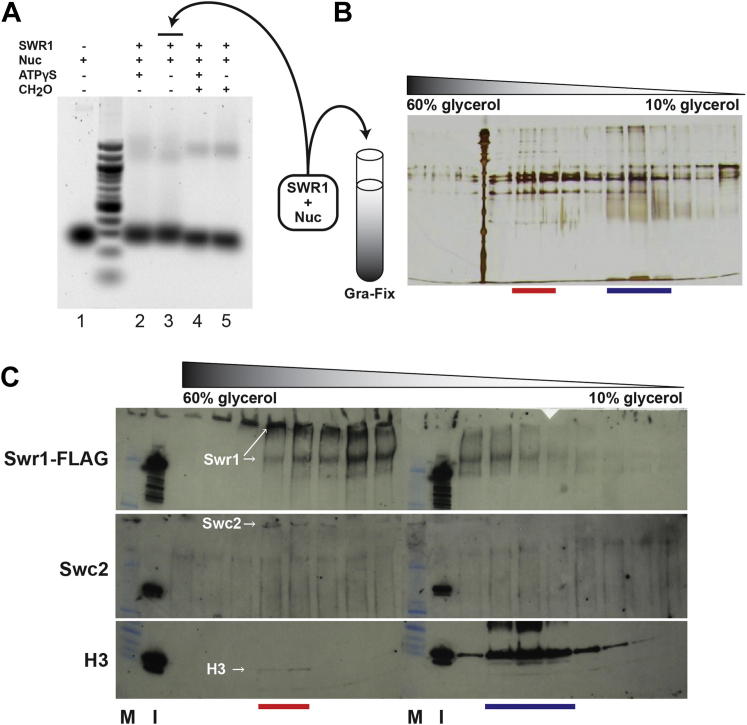
Assessment of Nucleosome Binding by SWR1 after GraFix Purification, Related to Figure 6 (A) Agarose gel electrophoresis of nucleosome-binding reactions in which SWR1 was incubated with recombinant nucleosomes. The effects of ATPγS and formaldehyde crosslinking on binding were also tested in this assay. After incubation, the reactions were resolved on a 1.3% agarose gel at 4°C in 0.2X TB buffer and stained with Sybr Green I. A free-nucleosome marker was run in Lane 1. (B) The SWR1 + nucleosome sample (equivalent to that in lane 3) was stabilized and purified through a GraFix gradient. The content in each fraction was analyzed by crosslink reversal and SDS-PAGE. (C) Western blot was performed to identify H3, Swc2, and Swr1-FLAG in select GraFix fractions. Due to incomplete reversal of formaldehyde crosslinking, some of the large subunits Swr1 and Swc2 were immobilized at the bottoms of the wells. Red and blue horizontal lines mark peaks for nucleosome-bound SWR1 and free nucleosomes, respectively. M: molecular weight marker. I: GraFix Input.

Our 3D reconstruction of SWR1 does not show a central cavity that could accommodate a nucleosome, as has been observed for the RSC complex (Chaban et al., 2008; Leschziner et al., 2007). To explore the possibility that SWR1 interacts with its substrate nucleosome in a different manner, we obtained the structure of SWR1 bound to a nucleosome. We used a nucleosome with a single 43 bp linker based on a characterization of the effect of linker length on nucleosome binding by SWR1 (Ranjan et al., 2013). Preliminary gel-shift studies showed similar binding of SWR1 to the nucleosome in the presence or absence of ATP (Ranjan et al., 2013). Therefore, we carried out an in vitro nucleosome-binding reaction in the absence of nucleotide (Figure S6A) and purified the sample using GraFix (Figure S6B). Western blotting confirmed the cosedimentation of histones with SWR1 in the glycerol gradient (Figure S6C), and the sample exhibited retarded electrophoretic mobility on a native gel relative to apo-SWR1 that had been similarly purified (Figure 6A). This suggested that a majority of the SWR1-nucleosome sample contained nucleosome-bound complexes. We imaged this sample under cryo-NS conditions.

**Figure S7:**
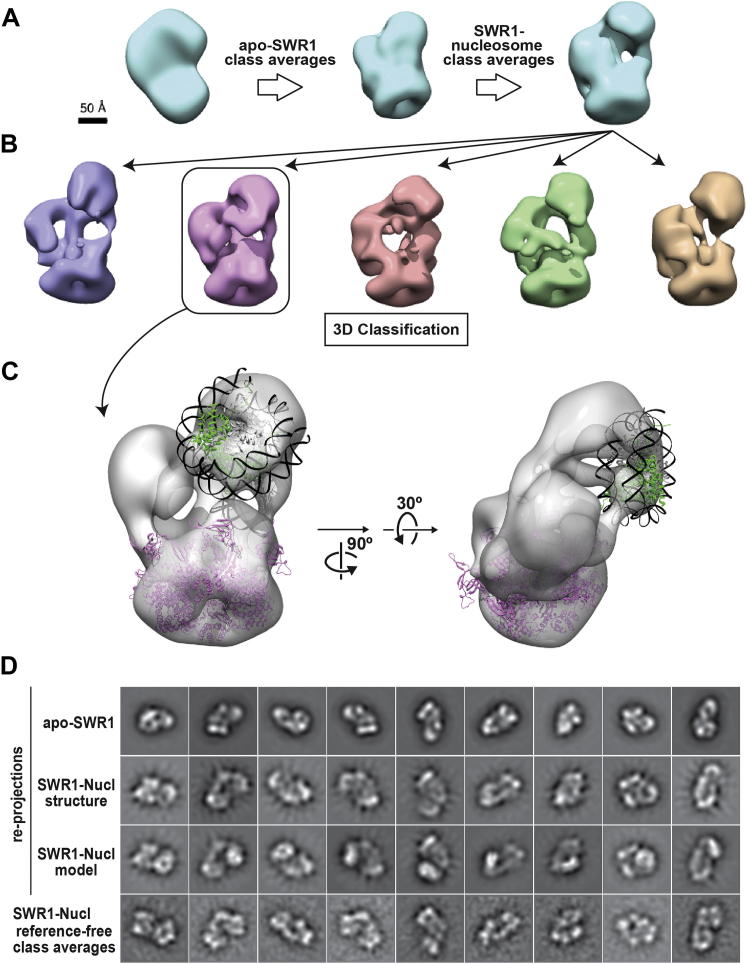
Generation of the 3D Cryo-Negative EM Structure of Nucleosome-Bound SWR1, Related to Figure 6 (A) The initial 3D reconstruction of SWR1-nucleosome was generated using a strategy analogous to that used for the apo-SWR1 structure. The OTR initial model (left) was first refined by projection matching against reference-free class averages generated from untilted, cryo-NS images of apo-SWR1. The resulting structure (middle) was filtered to 60Å and refined by projection matching against reference-free class averages generated from untilted, cryo-NS images of SWR1-nucleosome. The final refined structure is shown to the right. (B) The 3D map obtained (A) was used as the starting model to classify the SWR1-nucleosome data into five different reconstructions using the RELION software package (Scheres, 2012a). This panel shows the final five reconstructions generated by 3D classification. The second-from-left model was selected for single-model refinement against single particles assigned to the class. (C) 3D reconstruction after single-model refinement. Docking of the Rvb1/2 homology model and yeast nucleosome atomic models (White et al., 2001) into the 3D map of SWR1-nucleosome. H2A/H2B dimers are in green; H3/H4 in gray and the DNA in black. (D) Comparison of experimental reference-free class averages from the SWR1-nucleosome data to corresponding reprojections from 3D maps of apo-SWR1, SWR1-nucleosome and a 3D model generated by adding a docked nucleosome density into the SWR1-nucleosome map.

To obtain the 3D reconstruction of the SWR1-nucleosome complex, we first refined our apo-SWR1 model, obtained by refining the OTR model against 2D class averages of apo-SWR1, against 2D class averages generated from the SWR1-nucleosome data (Figure S7A). Using the resulting 3D map as a starting model, we performed maximum likelihood-based 3D classification (Scheres, 2012a) of the entire single-particle SWR1-nucleosome data set. We obtained five classes that displayed overall structural similarity (Figure S7B) and proceeded to further refine the one in which all modules could be most easily identified. The resulting 3D structure had a resolution of 34 Å (Figures 6C and 6E).

The SWR1-nucleosome structure is elongated relative to apo-SWR1 along an axis perpendicular to the Rvb1/Rvb2 ring (Figures 6B–6E). This elongation appears to be the result of an extension, away from the Rvb1/Rvb2 ring, of Swr1 and the C-Module (Figures 6B and 6D). Difference maps calculated between the apo-SWR1 and SWR1-nucleosome structures support this conformational change (Figures 6F and 6G), as does a comparison between reprojections of the apo-SWR1 3D map with reference-free class averages of the SWR1-nucleosome data (Figures 6I and S7D). At the reported resolution, we did not observe significant changes in the Rvb1/Rvb2 ring; Swr1 and the C-Module are the major densities that differ in positions between the two structures (Figures 6F and 6G). We note that the sample used in this analysis was purified and imaged under identical conditions to nucleosome-free SWR1. Therefore, experimental and computational variations are unlikely to have contributed to the observed conformational difference.

### SWR1 Engages the Nucleosome Core Particle via the Catalytic Subunit Swr1

The SWR1-nucleosome reconstruction showed a new density that is contiguous with that of the core subunit Swr1 and extends toward the Rvb1/Rvb2 ring (Figure 6C and 6E). This protrusion from Swr1 is also observed in 2D class averages (Figures 6I and S7D) and coincides with a peak in the difference map calculated by subtracting apo-SWR1 from SWR1-nucleosome (Figures 6G and 6H). We docked a 3D map generated from the yeast nucleosome crystal structure (White et al., 2001), filtered to the resolution of the SWR1-nucleosome map (34 Å), into the EM density. The extra density in our 3D map could accommodate the bulk of the nucleosome (Figures 6J and S7C). However, this density was not fully resolved, likely due to heterogeneity, both conformational and biochemical, in the data. The 3D map also indicated that the ATPase-containing portion of Swr1 mediates the most significant contact between SWR1 and the nucleosome core particle (Figures 6K and S7C).

To confirm the orientation of the bound nucleosome suggested by our data, we generated a model by computationally adding the density of a nucleosome into its putative density in our SWR1-nucleosome reconstruction (Figure 6J). We then compared reprojections from this composite model against experimental 2D class averages. This analysis indicated that the location and orientation of the modeled nucleosome were in general agreement with the experimental data (Figures 6I and S7D). In this orientation, the nucleosome appears to bind over a central depression formed between the Swr1 ATPase and the Rvb1/Rvb2 ring. One side of the octamer faces the complex, whereas the other side is completely exposed (Figure S7C).

## Discussion

### Functional Modules Assemble as Structurally Discrete Entities in SWR1

**Figure 7:**
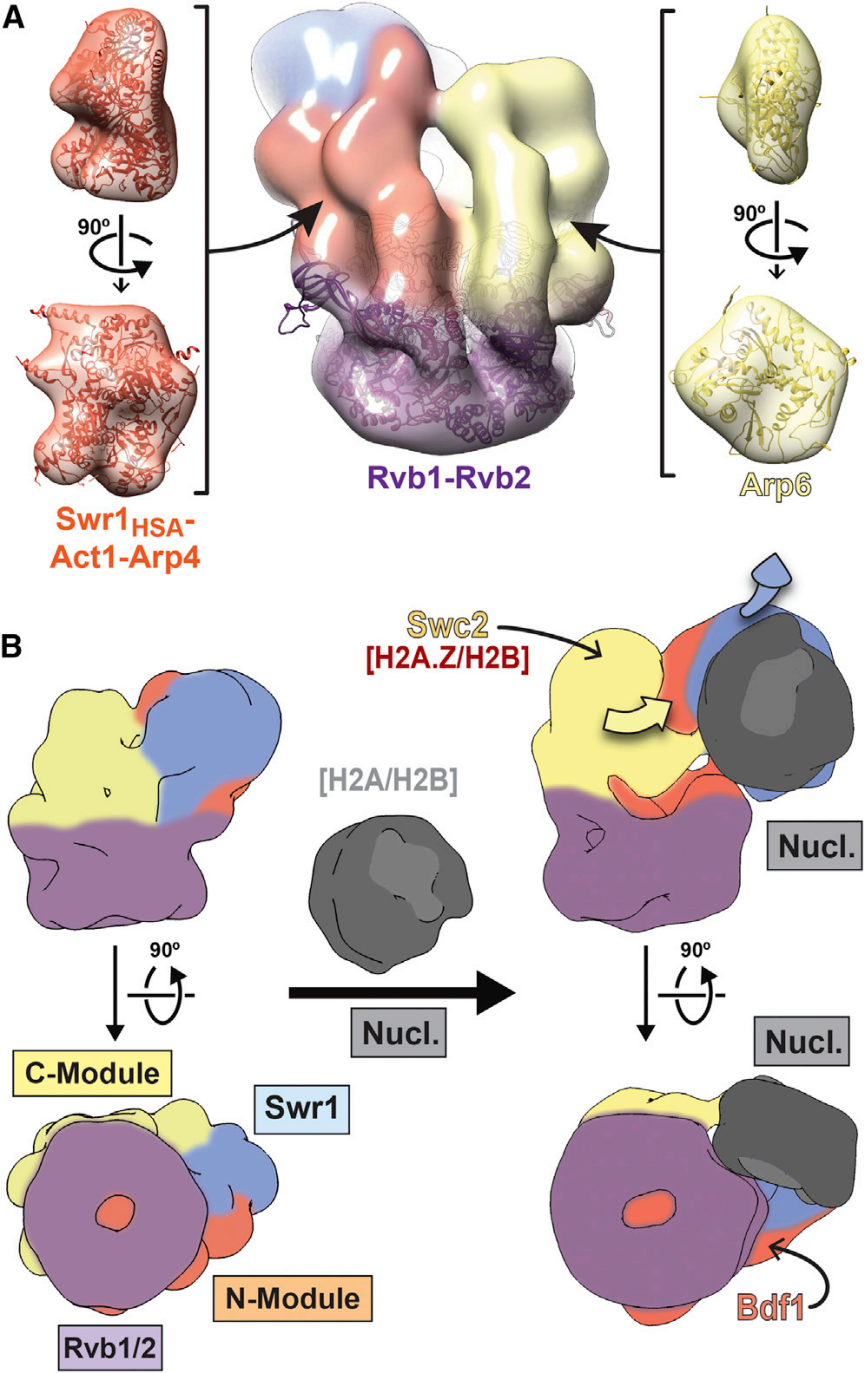
Molecular Architecture of SWR1 and Its Interaction with the Nucleosome (A) The SWR1 reconstruction, colored to highlight the different functional modules, is shown in the center with the Rvb1/Rvb2 homology model docked into the density. Known structures for homologs of SWR1 components are shown to highlight their compatibility with the structural features of the SWR1 EM map. The left side shows the crystal structure of Snf2HSA-Arp7-Arp9-Rtt102 (PDB 4I6M; Schubert et al., 2013) inside a density representing the structure at 28 Å resolution as a proxy for Swr1HSA-Act1-Arp4. The right side shows the crystal structure of yeast Arp4 (PDB 3QB0; Fenn et al., 2011) inside a density representing the structure at 28 Å resolution as a proxy for Arp6. (B) Schematic representation of the conformational changes observed in this work between apo-SWR1 and SWR1-nucleosome. Apo-SWR1 (left) and SWR1-nucleosome (right) are shown from two different orientations. The four functional modules are labeled at the bottom left and follow the same color conventions used throughout the paper. The arrows (top right) indicate the major conformational changes observed upon addition of nucleosome to SWR1. The nucleosome is shown in gray with the H2A/H2B dimers in a lighter shade. The approximate locations of subunits involved in binding to the H2A.Z/H2B dimer that will be inserted (Swc2 in the C-Module) and in binding to acetylated histone tails (Bdf1 in the N-Module) are indicated.

Our study revealed the structure of SWR1 to be composed of structurally discrete domains, with the substrate-binding N- and C-Modules arranged side-by-side and bracketed by the catalytic Swr1 ATPase and the Rvb1/Rvb2 ring (Figure 4D). We mapped the ATPase-containing bulk of the core subunit Swr1 to a location distal to the ring, where its position appears to be supported mainly by the N-Module (Figure 4D). Consistent with this arrangement, the SWR1-ΔN-Mod subcomplex exhibited more widespread structural changes than SWR1-ΔC-Mod, with changes in the Rvb1/Rvb2 ring detected in the 2D difference maps (Figure 4C). Our CX-MS data indicate that Arp4 mediates the N-module’s interaction with the Rvb1/Rvb2 ring (Figures 5 and 7A). ATP binding by Arp4 has been shown to regulate its association with macromolecular complexes in vivo (Sunada et al., 2005) and may play a regulatory role in the assembly of SWR1.

Our structural analysis of the subcomplexes provided direct visualization of the independent association of the N- and C-Modules with SWR1, as suggested by previous biochemical studies (Wu et al., 2009). In the absence of each ∼300 kDa module, the remaining components still associated in a manner similar to that in the full complex (Figures 4, S5B, and S5D). Therefore, SWR1 may assemble in a modular fashion that involves stable, preformed functional subcomplexes, as suggested for INO80 (Kapoor et al., 2013) and the histone acetyltransferase SAGA (Chittuluru et al., 2011). We speculate that modular assembly allows for efficient sharing of stable modules among functionally related complexes, thus regulating their recruitment and collective activity. This phenomenon may occur in SWR1, INO80, and the histone acetyltransferase NuA4—three complexes that are recruited to promoters and converge functionally at H2A.Z (Altaf et al., 2010; Mizuguchi et al., 2004; Papamichos-Chronakis et al., 2011) and compositionally at the Arp4-Act1 dimer (Kobor et al., 2004).

### The Rvb1/Rvb2 Ring Provides an Assembly Platform that Connects All Functional Modules in the Complex

We determined structurally and biochemically that Rvb1 and Rvb2 associate with the SWR1 complex as a single heterohexameric ring (Figure 2) and established the orientation of the ring relative to the rest of the complex (Figures 2D, 2H, and S3). The Rvb inserts, known to be involved in ring-ring interactions in dodecameric structures (Gorynia et al., 2011; López-Perrote et al., 2012; Torreira et al., 2008), face the core of SWR1, making them unavailable to interact with a second ring. Our biochemical quantitation of the Rvb1:Rvb2:Swr1 stoichiometry is in agreement with the 3:3:1 ratios indicated by the structural and proteomic data (Figure 3). Although ∼6:6:1 ratios have previously been reported for the highly related INO80 complex (Kapoor et al., 2013; Shen et al., 2000), suggesting the presence of two hexameric rings, our quantitation for INO80 also resulted in ∼3:3:1 stoichiometry (Figure S4). Therefore, we conclude that remodelers in the INO80 subfamily are characterized by the presence of a single hexameric Rvb1/Rvb2 ring.

Our results modify the previous assembly map of SWR1 (Figure 1A), which placed Swr1 at the center of the complex, bringing together individual modules via separate domains (Wu et al., 2005,2009). We now show that Swr1 adopts a peripheral position in the complex, with the catalytic ATPase spatially separated from the Rvb1/Rvb2 ring (Figure 4D). Their interaction across the complex may be supported by the long insert within the Swr1 ATPase (Wu et al., 2005). We found that the N-terminal HSA domain in Swr1 and the HSA-associated Arp4 subunit directly crosslinked to Rvb1 (Figures 5A and 5B). This indicates that the N-terminal half of Swr1 extends across the complex and may mediate interactions between the N-Module and the Rvb1/Rvb2 ring. We propose that the hexameric ring plays an important structural role as an assembly platform for the independently assembled, substrate-interacting N- and C-Modules. Their side-by-side arrangement may in turn be important for proper spatial coordination of the nucleosome and the H2A.Z/H2B dimer during dimer exchange.

Our data provide a structural view of a dimer exchanger as a compact and interconnected assembly of discrete functional modules. In such assembly, potential nucleotide- or substrate-dependent conformational changes in the catalytic ATPase (Lewis et al., 2008) and/or the hexameric ring (Gribun et al., 2008) could efficiently propagate and thus mediate global structural dynamics involved in dimer exchange.

### Nucleosome Binding by SWR1

Conformational changes have been reported for nucleosome-bound remodelers (Gangaraju et al., 2009); however, they have yet to be visualized. Our 3D structure of a SWR1-nucleosome cocomplex, together with the annotated 3D map of SWR1 alone, allowed us to characterize the extension of Swr1 and the C-Module, away from the Rvb1/Rvb2 ring, that occurs upon substrate binding (Figure 6). This extension was observed in the absence of nucleotides, suggesting that recognition of nucleosomal features, such as linker DNA, nucleosomal DNA, and/or unmodified histone tails, by various components of SWR1 is sufficient to promote this conformational change.

We showed that SWR1 makes only limited contact with the nucleosome, mediated primarily by the Swr1 ATPase, in contrast to other large remodelers that interact extensively with their substrate. The RSC complex, for example, envelops the nucleosome within a central cavity (Chaban et al., 2008; Saha et al., 2005), and the SWI/SNF complex contacts ∼50 bp, or nearly one gyre, of nucleosomal DNA (Dechassa et al., 2008). We speculate that relatively extensive substrate interactions may be a feature of remodelers that slide the histone octamer. Specifically, as nucleosomal DNA is unraveled from the octamer, these interactions may serve to prevent substrate disassembly. SWR1, however, is the only known remodeler that does not slide the octamer (A.R. and C.W, unpublished data) and shows very limited ATP-dependent mobilization of nucleosomal DNA (Jónsson et al., 2004; Papamichos-Chronakis et al., 2011). Therefore, chromatin remodeling by SWR1 may not require large-scale disruptions of histone-DNA contacts, and its limited interaction with the nucleosome may reflect this mechanistic distinction.

Our results do not address contacts that the complex may make with linker DNA, as observed for SWI/SNF (Dechassa et al., 2008), ISWIs (Dang and Bartholomew, 2007; Yamada et al., 2011), and suggested for the SWR1-related INO80 complex (Udugama et al., 2011). Unmodified histone tails may also interact with components of the N-Module, such as Swc4 and Arp4 (Boyer et al., 2004; Galarneau et al., 2000). All of these potential interactions, which would occur outside of the nucleosome core particle, could stabilize the substrate-bound conformation revealed by our 3D map. Furthermore, because the 3D map of nucleosome-bound SWR1 was obtained in the absence of nucleotides, it remains to be seen how ATP binding and/or hydrolysis by Swr1 and perhaps Rvb1/Rvb2 may further affect the overall structure of the substrate-bound complex. It has been shown that, whereas the ATPase activity of SWR1 is enhanced upon binding to a canonical nucleosome, which we used in our study, it achieves the highest level of stimulation when the second substrate, the H2A.Z/H2B dimer, is also bound (Luk et al., 2010). Structural characterization of this fully liganded and highly activated cocomplex should provide important new insights into SWR1 function.

In conclusion, our study presents the 3D structure of SWR1, revealing an interconnected assembly of discrete functional modules. This is also among the first structural characterizations of the functionally diverse AAA+ proteins Rvb1 and Rvb2 in the context of a larger complex. In SWR1, they form a single heterohexameric ring and serve as an assembly platform that connects all functional modules within the complex. Finally, we showed that SWR1 undergoes a large conformational change upon nucleosome binding (Figure 7B). The limited interaction between the complex and the nucleosome is distinct from other remodelers and may reflect unique mechanistic aspects of the dimer-exchange reaction.

## Experimental Procedures

### Sample Preparation

SWR1 was affinity purified from *S*. *cerevisiae* as described by Luk et al. (2010) (see Table S1 for strain information). Nucleosome-bound SWR1 was obtained by incubating 40 pmol recombinant nucleosomes with 10 pmol SWR1 in a 120 μl reaction for 30 min at room temperature. Apo- and nucleosome-bound SWR1 samples were further purified using the GraFix method (Kastner et al., 2008; Stark, 2010), substituting glutaraldehyde with formaldehyde.

For initial model reconstruction, SWR1 was applied to glow-discharged holey-carbon grids coated with a continuous-carbon support for ∼30 min at 4°C. The sample was stained with a 2% uranyl formate solution and dried under N_2_ gas. For refinement, apo-SWR1 and nucleosome-bound samples were absorbed onto glow-discharged Cu 200-mesh Quantifoil grids with a continuous-carbon support. Staining was carried out as described above, but stained samples were frozen in liquid N_2_ immediately after blotting, producing cryo-NS samples (De Carlo and Stark, 2010).

### EM Data Collection

Images were collected at +45° and −45° using a Tecnai G2 Spirit microscope (FEI, Hillsboro) operating at 120 keV and, equipped with a US4000 4 × 4k CCD camera (Gatan, Pleasanton) at a nominal magnification of 68,000×. The pixel size at the sample level was 1.65 Å.

Cryo-NS data were collected at liquid nitrogen temperature using a field-emission gun (FEG) Tecnai F20 transmission electron microscope (FEI) operating at 120 keV and equipped with a Gatan 4 × 4k CCD. Images were collected at a nominal magnification of 62,000× and an electron dose of ∼20 electrons/Å^2^. The pixel size at the sample level was 1.73 Å.

### Data Processing

Single particles were manually selected from micrographs using the Boxer interface in EMAN1 (Ludtke et al., 1999). We identified tilt mates using a custom-built SPIDER (Frank et al., 1996) script. Tilted particles were CTF corrected using the CTFTILT program (Mindell and Grigorieff, 2003). Untilted, cryo-NS particles were phase flipped in EMAN2 (Tang et al., 2007). Reference-free alignment and classification were carried out in IMAGIC (van Heel et al., 1996). Initial models were obtained using the OTR method (Leschziner, 2010).

We performed initial projection-matching refinement of the OTR models using class averages obtained from cryo-NS apo-SWR1 data. Refinement was carried out in SPIDER (Frank et al., 1996) using the AP SH and BP 32F functions. The resulting 3D maps were further refined against single cryo-NS particles of apo-SWR1 to obtain the final SWR1 reconstruction, and against reference-free class averages generated from cryo-NS data of SWR1-nucleosome. The latter 3D map was used as an initial model for 3D classification in the RELION program (Scheres, 2012b). Five 3D classes were generated, and one was further refined against single particles assigned only to that class using the “Autorefine” function in RELION.

### Isotopic Crosslinking-Mass Spectrometry

We crosslinked ∼45 μg of sample with 1 mM disuccinimidyl suberate d0/d12 (DSS; Creative Molecules) at 37°C for 30 min and subsequently quenched the reaction by adding ammonium bicarbonate. Trypsin digestion was carried out at 37°C overnight. We enriched for crosslinked peptides using size exclusion chromatography (Leitner et al., 2012), followed by LC-MS/MS analysis on an Orbitrap Elite mass spectrometer (Thermo Scientific, San Jose). Data analysis was performed using XQuest (Rinner et al., 2008).

Extended Experimental Procedures **Sample Purification**

SWR1 was affinity-purified from *S. cerevisiae* as previously described (Luk et al., 2010). We prepared a glycerol-formaldehyde gradient in a 4 ml ultracentrifuge tube (Beckman Ultra-Clear™) by layering 2 ml of “SWR1 buffer” (25 mM HEPES-KOH, pH 7.6, 1 mM EDTA, 2 mM MgCl_2,_ 0.01% NP-40, 1 mM DTT, 100 mM KCl) containing 10% glycerol and 0.2% formaldehyde over 2 ml of SWR1 buffer containing 60% glycerol and 1.5% formaldehyde. The gradient was formed using a GradientMaster instrument (Model 107ip, BioComp). After applying 4-10 pmoles of SWR1 to the top of the gradient, we centrifuged the sample in a SW 60 Ti rotor (Beckman) at 35,000 rpm and 4°C for 20 hr. We then manually fractionated the gradient from bottom to top into 100 μl fractions and quenched the formaldehyde in each fraction using 80 mM glycine. We stored the fractions at 4°C.

To determine the best fraction to image, we reversed the crosslinks for 15 μl of each fraction in 0.3 M Tris, 0.1% SDS, and β-mercaptoethanol for 12 hr at 65°C and 30 min at 95°C in a thermocycler (Jackson, 1999). We screened for fractions containing the full complement of 14 subunits by running the treated samples in a 10% SDS-PAGE gel and silver-staining it. Having identified candidate fractions after crosslink reversal and SDS-PAGE analysis, we proceeded to dialyze the original crosslinked samples in those fractions against SWR1 buffer containing no glycerol. The dialyzed samples were stored at 4°C, ready to be stained and imaged.

We prepared mono-nucleosomes containing recombinant fly H3-H4, yeast H2A-H2B and 190 bp DNA bearing the 601 nucleosome-positioning sequence and a 43 bp asymmetric linker. To obtain nucleosome-bound SWR1, we carried out an in vitro binding reaction with 40 pmoles reconstituted nucleosomes and 10 pmoles SWR1 in a 120 μl reaction for 30 min at room temperature. We then proceeded to purify SWR1-Nucl as described above for SWR1.

**Native Gel Electrophoresis and Western Blot**

**Native Gel Electrophoresis**

To qualitatively assess nucleosome occupancy in the GraFix-treated sample, we compared the electrophoretic mobility of this sample against that of apo-SWR1, which was similarly purified. After dialysis, crosslinked samples from single fractions were electrophoresed in a NuPAGE Novex 3%–8% Tris-Acetate, 1.0 mm gel (Invitrogen) at 4°C. Sample loading buffer contained 10% glycerol, 0.01% w.v. bromophenol blue, 43 mM imidazole, 35 mM HEPES, pH 7.4. Electrophoresis buffer contained 43 mM imidazole and 35 mM HEPES, pH 7.4. The gel was silver-stained.

**Western Blot**

To confirm cosedimentation of nucleosomes and SWR1 in the glycerol gradient, we quenched and reversed the formaldehyde crosslinking as described above. Then, an aliquot of each fraction was electrophoresed in a precast PROTEAN 4%–20% polyacrylamide gel (Bio-Rad). Proteins were transferred onto a nitrocellulose membrane and blotted for,

• H3 using rabbit α-H3 (AB1791) at 1:3,000 dilution and GαRb 2° at 1:5,000 dilution
• Swr1-3xFLAG using mouse α-FLAG (Bio-Rad) at 1:3,000 dilution and rabbit anti-mouse 2° at the same dilution
• Swc2 using chicken α-Swc2 (Wu et al., 2009) at 1:10,000 dilution and α-chicken HRP at 1:5,000 dilution

Membranes were exposed to Kodak chemiluminescence films.

**Electron Microscopy**

**Sample Preparation**

We obtained the initial models of SWR1 using negatively stained samples. We applied 5-10 μl of the dialyzed sample to homemade holey grids coated with a thin layer of carbon, let the sample absorb for 15-30 min at 4°C, stained with a 2% uranyl formate solution and then floated a second layer of thin carbon (“sandwich”) before drying the grid under a gentle flow of N_2_ gas.

The initial models were refined against cryo-negative stain (cryo-NS) data. To prepare cryo-NS samples, we stained as described above but, instead of drying, we froze the stained grids in liquid nitrogen (De Carlo and Stark, 2010).

**Imaging**

To build the initial 3D models for SWR1, we collected images under low-dose conditions at +45° and −45° in a Tecnai G2 Spirit microscope (FEI, Hillsboro, OR), operating at 120 keV and equipped with a US4000 4k × 4k CCD camera (Gatan, Inc, Pleasanton, CA) at a nominal magnification of 68,000 and with a dose of ∼20 electrons/Å^2^. The pixel size at the sample level was 1.65Å.

To obtain apo- and nucleosome-bound SWR1 data for refinement, we collected untilted cryo-NS data at liquid-nitrogen temperature and under low-dose conditions. We used a field-emission gun (FEG) Tecnai G2 F20 transmission electron microscope (FEI) operating at 120 keV and equipped with a Gatan 4k × 4k CCD. Images were collected at a nominal magnification of 62,000x and an electron dose of ∼20 electrons/Å^2^. The pixel size at the sample level was 1.73 Å.

**Initial Model Generation**

In order to extract single particles from the micrographs we windowed out particles in one set of micrographs using the Boxer interface in EMAN1 (Ludtke et al., 1999) and used custom-built SPIDER (Frank et al., 1996) scripts to calculate alignment parameters between the +45° and −45° micrographs and extract the tilt mates in the other set (Leschziner, 2010). We estimated and corrected for the Contrast Transfer Function (CTF) using the program CTFTILT (Mindell and Grigorieff, 2003) and the SPIDER command TF CT. Single particles were binned by 2, resulting in a pixel size of 3.3 Å. We combined the +45° and −45° data sets into a stack of ∼32,000 particles and performed reference-free 2D alignment and classification in IMAGIC (van Heel et al., 1996). We computed initial models from classes containing 100–200 members using the Orthogonal Tilt Reconstruction approach as described (Leschziner, 2010).

**Projection-Matching Refinement**

We initially refined the OTR models against class averages of cryo-NS data. To generate the class averages, we extracted particles from the micrographs as described above and performed CTF estimation and phase flipping using the EMAN2 workflow (Tang et al., 2007). Then, the particles were binned by 2, resulting in a pixel size of 3.45 Å. We subjected ∼32,000 particles to reference-free 2D alignment and classification in IMAGIC (van Heel et al., 1996). In order to minimize heterogeneity, we generated classes with relatively few (15-20) particles.

We filtered the OTR models to 80 Å resolution and performed 15-23 iterations of projection matching refinement using angular steps of 25°, 20°, 15°, 10°, and 8°-5° (every degree) against 2D class averages in SPIDER (Frank et al., 1996) using the AP SH and BP 32F commands. To minimize build up of noise in the reconstructions, we applied a threshold mask calculated for 500% to 150% the theoretical molecular weight of the sample (1.0 MDa). The mask was gradually tightened during refinement and its filtration was determined by the resolution of the 3D map, computed according to the 0.5 FSC criterion. Refinement results were generally stable after 15 iterations, and the resolutions of the 3D maps were 50-60 Å. We then refined the resulting 3D maps (without additional filtration) against single cryo-NS particles. For this step, we carried out 15 iterations of projection-matching refinement at angular steps 25°, 21°, 18°, 15°, 13°, 11° and 10°–4° (every degree). Threshold masks computed for 500% to 100% the MW were also utilized as described above.

**Alignment of 2D Images**

To compare 3D maps against experimental data, we generated reprojections of the filtered maps at defined theta values using the PJ 3Q command in SPIDER (Frank et al., 1996). The reprojections and experimental class averages were aligned to each other using the AP SH and RT SQ commands in SPIDER (Frank et al., 1996).

**Visualization of and Docking into 3D Maps**

We performed 3D structure analysis and image rendering using the UCSF Chimera package (Pettersen et al., 2004). EM-like 3D maps were generated from published crystal coordinates using the CP FROM PDB command in SPIDER (Frank et al., 1996). To dock 3D maps or crystal structures into our EM densities, we roughly placed the former into the latter and used Chimera’s “Fit in Map” function for the final fitting.

To generate the composite nucleosome-bound SWR1 map, we generated a 3D map of the yeast nucleosome from the published crystal coordinates (PDB: 1IDB, [White et al., 2001]) as described above. Then, we placed this map into our experimental map and converted the former’s 3D coordinate system to the latter via resampling (*vop resample* command in Chimera). The two maps were then normalized, added, and filtered in SPIDER (Frank et al., 1996).

**Segmentation of the SWR1 3D Map**

We segmented the SWR1 3D map using the Eraser tool in Chimera. We used the results from our module mapping (Figure 4) to guide the segmentation. Once we had obtained a segmented module using the eraser, we calculated the molecular weight of the enclosed volume by obtaining the number of voxels in Chimera.

**Difference Mapping**

We performed 2D difference mapping between class averages by first aligning, rotating, and shifting them in IMAGIC (van Heel et al., 1996) using the MSA-ALIGN command. Then, we normalized and subtracted the aligned images in SPIDER (Frank et al., 1996).

The difference maps were normalized (mean = 0, standard deviation = 1). Threshold masks were calculated for each of them, setting values below 2σ to 0 and these masks were then applied to the normalized difference maps. The resulting images were colored in Photoshop by turning them into RGBs and converting the grayscale values to a rainbow gradient. The gradient begins at 2σ (as determined by the thresholding) and ends at around 6σ, based on the maximum pixel values present in each of the difference maps. These maxima changed slightly between difference maps, ranging from ∼6σ to ∼7σ. We used a single color scale for the figure for simplicity, even though it ignores small differences in the heights of the peaks among the images shown.

**3D Classification of SWR1-Nucleosome Reconstructions**

To obtain the 3D structure of nucleosome-bound SWR1, we used the 3D map obtained after refinement of the OTR model against apo-SWR1 class averages. This model was low-pass filtered to 60 Å and refined against 2D class averages generated from cryo-NS data of the nucleosome-bound sample. The SWR1-nucleosome class averages were obtained as described above for apo-SWR1. Using the resulting 3D map as a starting model, we performed maximum-likelihood-based 3D classification (Scheres, 2012b) using the RELION program (Scheres, 2012a). We generated 5 classes from ∼45,000 single particles. Single particles were phase-flipped in EMAN2 (Tang et al., 2007), binned by 3 for a resulting pixel size of 5.17 Å, and normalized in XMIPP (Sorzano et al., 2004). After 3D classification, we performed single-model refinement by using the “Autorefine” option in RELION. Because a majority of the resulting 3D maps exhibited missing densities, we selected a model in which densities for all modules identified in the apo-SWR1 structure were accounted for. To minimize heterogeneity, we refined it against single particles assigned only to that class by 3D classification, which constituted ∼20% of the data set.

**Homology Model Generation**

The 3D homology model of *S. cerevisiae* Rvb1/2 heterohexamer was computed using the SWISS-MODEL interface (Bordoli and Schwede, 2012). We used the crystal structure of the human Rvb1 homolog (2C9O, [Matias et al., 2006]) as the template to align sequences for *Saccharomyces cerevisiae* Rvb1 (YDR190C) and Rvb2 (YPL235W). With the 3D homology models for the Rvb1 and Rvb2 monomers, we generated the hetero-hexameric Rvb1/2 model in PyMOL (The PyMOL Molecular Graphics System, Version 1.5.0.4 Schrödinger, LLC) by computationally aligning each monomer to the corresponding homolog in the crystal structure of the truncated human Rvb1/2 hexameric ring (2XSZ, [Gorynia et al., 2011]). The composite final structure was saved as a PDB file. Subsequent analyses involving this homology model were carried out in UCSF Chimera (Pettersen et al., 2004).

**Stoichiometry Quantification**

Purified SWR1 and INO80 complexes were resolved on 4%–12% Bis-Tris gel (Novex) with MOPS running buffer. Protein gels were stained with Coomassie or Sypro Orange dye and imaged on Fuji Image Quant LAS 3000. Intensity of relevant protein bands was measured using ImageQuant TL software (GE). The ratios of Swr1 or Ino80 to Rvb1 and Rvb2 were calculated after normalizing the band intensities for protein size.

We approximated Rvbs:Swr1 stoichiometry in the GraFix-treated sample for each fraction. After crosslink reversal and SDS-PAGE, as described above, the gel was silver-stained and imaged on the GE (Amersham) Typhoon Trio Imager. Band intensities were measured using the ImageQuant TL software (GE) after background subtraction (rolling-ball method).

**Chemical Crosslinking Coupled to Mass Spectrometry**

We crosslinked roughly 45 μg of sample with 1 mM disuccinimidyl suberate d0/d12 (DSS, Creative Molecules Inc.) directly in SWR1 buffer at 37°C for 30 min and subsequently quenched the reaction by adding ammonium bicarbonate to a final concentration of 50 mM for 10 min at 37°C.

We reduced the sample with 2.5 mM Tris (2-Carboxyethyl) phosphine hydrochloride (TCEP, Pierce) in 8 M urea at 37°C for 30 min and subsequently alkylated it with 5 mM iodoacetamide (Sigma-Aldrich) for 30 min at room temperature in the dark. For digestion, we diluted the sample with ammonium bicarbonate to a 1 M final concentration of urea and added 2% w/w trypsin (Promega). Digestion was carried out at 37°C over night and stopped by acidification to 2% (w/v) trifluoroacetic acid (TFA). We purified peptides with Sep-Pak C18 MicroSpin columns (Waters, Milford, MA), according to the manufacturer’s protocol, followed by enrichment of crosslinked peptides using size exclusion chromatography (Leitner et al., 2012). We carried out LC-MS/MS analysis on an Orbitrap Elite mass spectrometer (Thermo Scientific, San Jose, CA).

We searched the data using XQuest (Rinner et al., 2008) in iontag mode against a database containing the protein sequences of all 14 previously identified SWR1 proteins with a precursor mass tolerance of up to 20 ppm. For matching of fragment ions tolerances of 0.2 Da for common-ions and 0.3 Da for crosslink ions were used. We identified crosslinked peptides with a linear discriminant (ld) score > 25 and further analyzed them by visual inspection in order to ensure good matches of ion series on both crosslinked peptide chains for the most abundant peaks.

## Accession Numbers

The cryo-NS maps of SWR1 (EMD-5626) and the SWR1-nucleosome complex (EMD-5638) were deposited in the EM Data Bank.

## Supplemental Information

Movie S1. The Three-Dimensional Structure of the SWR1 Complex, Related to Figure 1 The movie shows the 3D reconstruction of SWR1 with 360o rotations around the X and Y axes. After those rotations, the structure is rendered with transparency to display the docked Rvb1/2 heterohexameric ring and is rotated again by 360o around the X and Y axes.

Document S1. Tables S1 and S2

Document S2. Article plus Supplemental Information
