# Supplementary material for: Molecular Architecture of the ATP-Dependent Chromatin-Remodeling Complex SWR1
Source: Cell. 2013 Sep 12;154(6):1220–31. doi: 10.1016/j.cell.2013.08.018 (PMC3776929; doi:10.1016/j.cell.2013.08.018)
Supplement: Document S1. Tables S1 and S2 [file mmc2.pdf]

Table S1. Yeast Strains Used in This Study, Related to the Experimental Procedures

SWR1-3Flag *htz1Δ*	*W1588C-4C swr1::SWR1-3Flag-P-KanMX-P htz1Δ::natMX4*	Kind gift of Wei-hua Wu
SWR1-2Flag (ΔN2)	*W1588-4C swr1::natMX [pRS416-swr1ΔN2-2Flag]*	Wei-hua et al 2006
SWR1-Flag arp6Δ	*W1588-4C SWR1-3Flag kanMX4 arp6::hphMX6 htz1::natMX*	Wei-hua et al 2006

This table lists the *S.cerevisiae* strains used in this study. The three strains were the sources of the SWR1 (top), SWR1-ΔN-Module (middle) and SWR1-ΔC-Module (bottom) complexes.

Table S2. Inter- and Intraprotein Crosslinks Identified, Related to Figure 5

INTERPROTEIN CROSSLINKS

Crosslinked peptides (Protein1-Protein2)	Protein1	Xlink1 (AA)	Protein2	Xlink2 (AA)	Cα-Cα (Å)	Id score
HQGIMVGMGQKDSYVGDEAQSKR-TKSNESR	|P60010|ACT1	K50	|P38326|SWC5	K205		37.31
LLAQAEDEDDVKAANLAMR-TKSNESR	|Q05471|SWR1	K1456	|P38326|SWC5	K205		33.74
KIIQERIR-SKGRSGSK	|Q03388|VPS72	K218	|P35817|BDF1	K504		33.21
GLGLDESGVAKRVEGGFVGQIEAR-FAVQAVKKR	|Q03940|RVB1	K42	|Q05471|SWR1	K418		32.2
FAPGFKVLTYYGSPQQR-EKGVK	|Q05471|SWR1	K772	|P31376|SWC3	K407		30.55
SAAEIAEEEALVVESKK-SSTKARIAR	|P53201|SWC4	K262	|Q05471|SWR1	K371		30.05
FINHLIKKALEPK-EIEKFKTK	|P80428|ARP4	K195	|P31376|SWC3	K498		29.43
HKEQESQHMLTQEER-KSIGIK	|Q03388|VPS72	K228	|Q12464|RVB2	K123		29.19
LLSSSGKVGSVLDGSKEAR-LENLVKQEAINGS	|P38326|SWC5	K138	|P53930|YAF9	K219		28.5
NDYVPLKR-KKYLQR	|P80428|ARP4	K323	|P53201|SWC4	K238		26.85
LLSSSGKVGSVLDGSKEAR-ETDSLQPITSKEIK	|P38326|SWC5	K138	|Q05471|SWR1	K326		26.64
STKILETSANYL-GVSKTR	|Q03940|RVB1	K454	|Q12464|RVB2	K331	14.5Å	26.33
HQGIMVGMGQKDSYVGDEAQSKR-TKSNESR	|P60010|ACT1	K61	|P38326|SWC5	K205		25.82
TAAHTHIKGLGLDESGVAKR-SDKKVTPTEEK	|Q03940|RVB1	K31	|P80428|ARP4	K335		25.1

INTRAPROTEIN CROSSLINKS

Crosslinked peptides (Protein1-Protein2)	Protein1	Xlink1 (AA)	Protein2	Xlink2 (AA)	|AA1 - AA2|	Cα-Cα (Å)	Id score
RAAGVILKMVQNGTIAGR-GVSKTR	|Q12464|RVB2	K59	|Q12464|RVB2	K331	272	33.6Å	44.18
IESESGGLIKTR-NKVDYSR	|P38326|SWC5	K79	|P38326|SWC5	K64	15		38.33
SNSGVVKTWR-NDYVPLKR	|P80428|ARP4	K313	|P80428|ARP4	K323	10	26.0Å	38.27
KSESAYAEQLLK-QRQEMQTALKR	|P53201|SWC4	K372	|P53201|SWC4	K370	2		38.01
STTAAQQEDKILIER-VGSVLDGSKEAR	|P38326|SWC5	K151	|P38326|SWC5	K138	13		37.34
STTAAQQEDKILIER-TKSNESR	|P38326|SWC5	K151	|P38326|SWC5	K205	54		37.08
SNSGVVKTWR-NDYVPLKR	|P80428|ARP4	K313	|P80428|ARP4	K323	10	26.0Å	36.76
SIIITTKSYNEQEIK-GVSKTR	|Q12464|RVB2	K357	|Q12464|RVB2	K331	26	23.9Å	36.65
AGLNDELVLHNKDGFLAR-VGSAEDERYKELR	|P38326|SWC5	K263	|P38326|SWC5	K285	22		36.61
GTNYKSPHGLPLDLLDR-GVSKTR	|Q12464|RVB2	K338	|Q12464|RVB2	K331	7	4.1Å	35.52
FGPSTNKKPFR-SYKGEMR	|Q12509|ARP6	K23	|Q12509|ARP6	K144	121		34.91
RKSESAYAEQLLK-QEMQTALKRK	|P53201|SWC4	K372	|P53201|SWC4	K370	2		34.13
LKTVVTYDMKR-DKKNK	|P35817|BDF1	K525	|P35817|BDF1	K520	5		33.06
IESESGGLIKTRR-NKVDYSR	|P38326|SWC5	K79	|P38326|SWC5	K64	15		32.75
VGSAEDERYKELR-TKSNESR	|P38326|SWC5	K285	|P38326|SWC5	K205	80		32.68
KDEEEQLKR-WNMAEKAYR	|Q05471|SWR1	K433	|Q05471|SWR1	K426	7		32.54
AGLNDELVLHNKDGFLAR-TKSNESR	|P38326|SWC5	K263	|P38326|SWC5	K205	58		32.54
FAVQAVKKR-WNMAEKAYR	|Q05471|SWR1	K418	|Q05471|SWR1	K426	8		32.1
KTVVHTVSLHEIDVINSR-ASGKITK	|Q12464|RVB2	K233	|Q12464|RVB2	K198	35	13.5Å	32.04
SNSGVVKTWR-FINHLIKK	|P80428|ARP4	K313	|P80428|ARP4	K195	118	31.5Å	31.37
SDIKRDETTNEDSDDQVR-	|Q03388|VPS72	K754	|Q03388|VPS72	K746	8		31.02

IQDPISKEEGR							
KSESAYAEQLLKDFNSDER-QRQEMQTALKR	|P53201|SWC4	K372	|P53201|SWC4	K370	2		30.66
FINHLIKK-KPEFIKK	|P80428|ARP4	K195	|P80428|ARP4	K218	23	19.5Å	30.04
GSKKR-IRKER	|P35817|BDF1	K497	|P35817|BDF1	K488	9		29.96
FAVQAVKKR-WNMAEKAYR	|Q05471|SWR1	K418	|Q05471|SWR1	K426	8		29.59
ILRKDEEEQLK-WNMAEKAYR	|Q05471|SWR1	K433	|Q05471|SWR1	K426	7		29.54
EKVLAGDVISIDKASGK-SITGGHKQGK	|Q12464|RVB2	K183	|Q12464|RVB2	K154	29	19.5Å	29.17
ETHLSLEERGEKFTDDVAK-KGTNGDLTR	|Q05471|SWR1	K74	|Q05471|SWR1	K82	8		28.88
HKKSLAR-KAKEEER	|Q05471|SWR1	K407	|Q05471|SWR1	K400	7		28.84
HKKSLAR-KAKEEER	|Q05471|SWR1	K407	|Q05471|SWR1	K400	7		28.34
WNMAEKAYRILR-KDEEEQLKR	|Q05471|SWR1	K426	|Q05471|SWR1	K433	7		27.87
GTNYKSPHGLPLDLLDR-GVSKTR	|Q12464|RVB2	K338	|Q12464|RVB2	K331	7	4.1Å	27.26
KVTPTEEKEQEAVSK-TKPSGVNKSDK	|P80428|ARP4	K336	|P80428|ARP4	K332	4	N/A	26.23
STKILETSANYL-LLFLDAKR	|Q03940|RVB1	K454	|Q03940|RVB1	K450	4	4.9Å	26.19
ADDENAEKQQSKEAK-SSKTAATEPEPKK	|P31376|SWC3	K380	|P31376|SWC3	K371	9		25.41
YGFAEELFLPKEDDIPANWPR-TLEETKTELSSTAKR	|P80428|ARP4	K296	|P80428|ARP4	K259	37	21.2Å	25.35

This table lists all the crosslinked species identified with an Id score > 25. The crosslinked peptides are shown with single-letter code, separated by a dash (first column). The crosslinked lysines are indicated in red and underlined. Columns 2 and 4 identify the proteins that gave source to the first and second peptides in the crosslinked species, respectively. Columns 3 and 5 indicate the positions of the crosslinked lysine residues in the full-length proteins for the first and second peptides in the crosslinked species, respectively. Column 6, which refers only to intraprotein crosslinks, indicates the distance, in residues, between the two crosslinked lysines. Column 7 shows the distance, in Å, between the α carbons of the crosslinked lysines whenever a crystal structure is available. The distance reported for the crosslink between Rvb1 and Rvb2 was measured using the homology model we generated for the *S.cerevisiae* heterohexamer (see Supplementary Methods). Column 8 reports the Id scores.
